# Emerging strategies to overcome resistance to third-generation EGFR inhibitors

**DOI:** 10.1186/s13045-022-01311-6

**Published:** 2022-07-15

**Authors:** Kunyu Shi, Guan Wang, Junping Pei, Jifa Zhang, Jiaxing Wang, Liang Ouyang, Yuxi Wang, Weimin Li

**Affiliations:** 1 Department of Respiratory and Critical Care Medicine, Targeted Tracer Research and Development Laboratory, Institute of Respiratory Health, Innovation Center of Nursing Research, Nursing Key Laboratory of Sichuan Province, West China Hospital, Sichuan University, Chengdu, 610041 China; 2 Department of Pharmaceutical Sciences, College of Pharmacy, University of Tennessee Health Science Center, Memphis, TN 38163 USA; 3 State Key Laboratory of Biotherapy and Cancer Center, West China Hospital, and Collaborative Innovation Center of Biotherapy, Sichuan University, Chengdu, 610041 China; 4 Precision Medicine Key Laboratory of Sichuan Province and Precision Medicine Research Center, West China Hospital, Sichuan University, Chengdu, 610041 China; 5 Tianfu Jincheng Laboratory, Chengdu, 610041 China

**Keywords:** Epidermal growth factor receptor (EGFR), Drug resistance, Inhibitors, Structure–activity relationship, Tyrosine kinase, Cancer

## Abstract

Epidermal growth factor receptor (EGFR), the receptor for members of the epidermal growth factor family, regulates cell proliferation and signal transduction; moreover, EGFR is related to the inhibition of tumor cell proliferation, angiogenesis, invasion, metastasis, and apoptosis. Therefore, EGFR has become an important target for the treatment of cancer, including non-small cell lung cancer, head and neck cancer, breast cancer, glioma, cervical cancer, and bladder cancer. First- to third-generation EGFR inhibitors have shown considerable efficacy and have significantly improved disease prognosis. However, most patients develop drug resistance after treatment. The challenge of overcoming intrinsic and acquired resistance in primary and recurrent cancer mediated by EGFR mutations is thus driving the search for alternative strategies in the design of new therapeutic agents. In view of resistance to third-generation inhibitors, understanding the intricate mechanisms of resistance will offer insight for the development of more advanced targeted therapies. In this review, we discuss the molecular mechanisms of resistance to third-generation EGFR inhibitors and review recent strategies for overcoming resistance, new challenges, and future development directions.

## Introduction

**Fig. 1:**
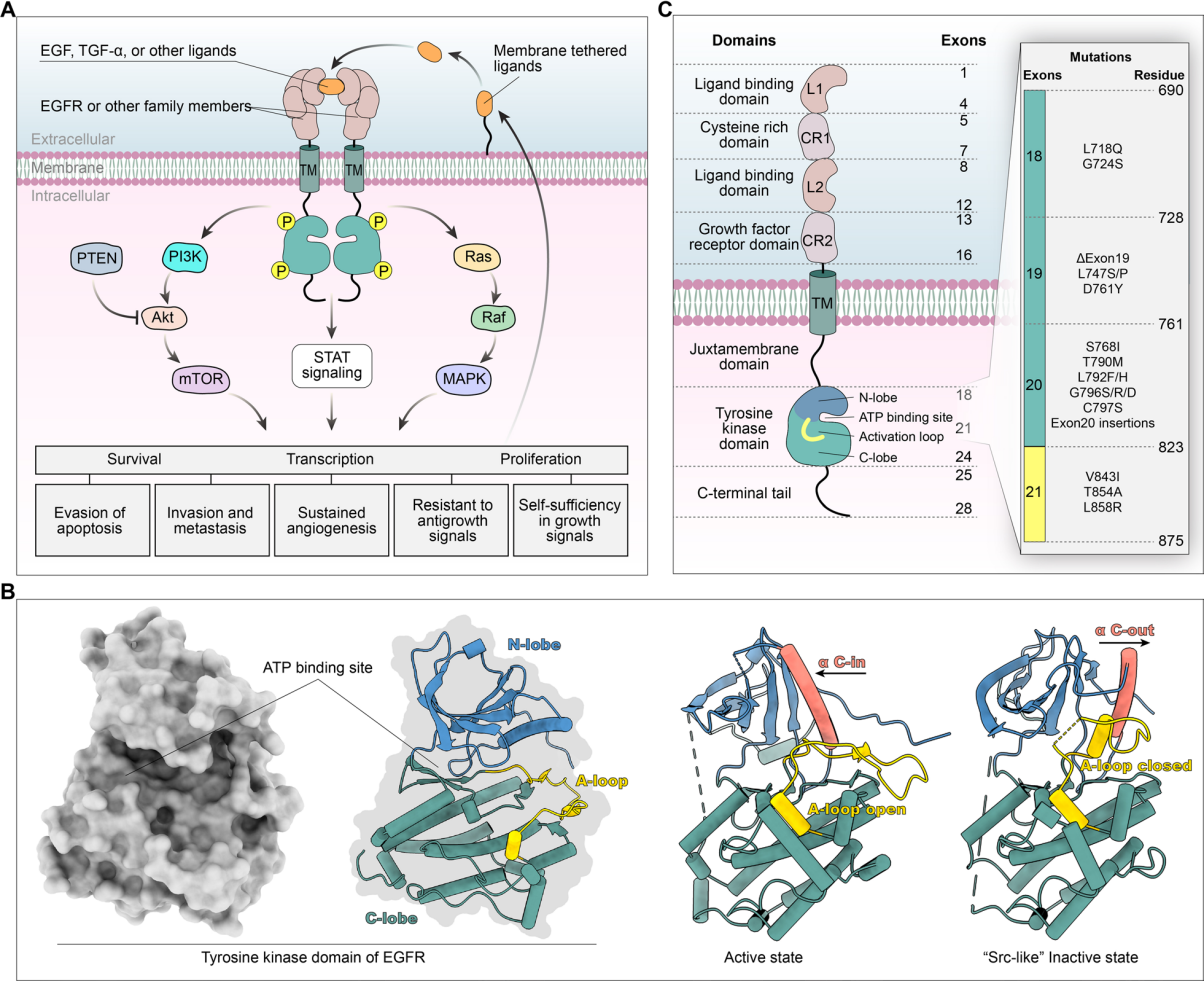
Structure and functions of EGFR. **A** EGFR exon boundaries and associated extracellular, transmembrane, and intracellular protein domains. EGFR is involved in multiple signaling pathways and regulates numerous cell functions. **B** The tyrosine kinase domain of EGFR and the activation or inactivation of the catalytic domain. **C** EGFR domains and the molecular mechanisms of acquired resistance. The intracellular domain contains a juxtamembrane domain, tyrosine kinase domain, and multiple C-terminal tyrosine residues. Multiple mutations within the tyrosine kinase domain are associated with resistance and sensitivity to EGFR-TKIs

Epidermal growth factor receptor (EGFR) is a member of the receptor tyrosine kinase (RTK) superfamily that consists of exon boundaries and associated extracellular, transmembrane, and intracellular protein domains. EGFR is involved in multiple signaling pathways and regulates numerous cell functions (Fig. 1A). This transmembrane glycoprotein is composed of a cysteine-rich extracellular ligand binding domain, hydrophobic transmembrane domain, cytoplasmic RTK domain, and C-terminal domain. The RTK domain contains an N-lobe consisting of five β-sheet strands and one αC helix and a C-lobe containing the main helices of a highly flexible activation loop (A-loop) [1]. The deep cleft at the junction of these two lobes forms the binding pocket for the adenine ring of ATP. The conformation of three conserved structural elements, namely the Asp-Phe-Gly (DFG) motif, αC helix, and A-loop, critically regulates the activation or inactivation of the catalytic domain. When EGFR is in the active state, the important catalytic residue D855 is located in the ATP binding site, stabilizing the ATP-loaded complex (DFG-in) and αC helix (αC-in). In the inactive state, EGFR forms a Src-like structure, including a closed A-loop, αC-out, and DFG-in [2]. (Fig. 1B). EGFR can dimerize upon binding by ligands, such as amphiregulin, β-cytokines, epidermal growth factor (EGF), heparin-binding EGF-like growth factor (HB-EGF), and transforming growth factor (TGF). The activation of the intracellular tyrosine kinase domain and autophosphorylation, which initiates the Ras/RAF/MEK, signal transducer and activator of transcription (STAT), PI3K/AKT/mTOR and other downstream signaling pathways, are closely related to embryonic development and stem cell division [2–4]. Overexpression of wild-type (WT) EGFR protein with or without EGFR gene amplification or a kinase-activating mutation further enhances cell proliferation, migration, survival, and antiapoptotic responses through signaling cascades, and these processes are closely related to the occurrence and development of many types of epithelial-derived cancer, such as non-small cell lung cancer (NSCLC), breast cancer, glioma, head and neck cancer, cervical cancer, and bladder cancer. Among these cancers, lung cancer appears to be the most common and has the characteristics of aberrant proliferation, metastasis, and drug resistance [5–8]. Thus, EGFR has become a promising target for anticancer drug design and development. EGFR tyrosine kinase inhibitors (EGFR-TKIs) have achieved remarkable results in the clinic [9]. However, most patients develop acquired drug resistance to first- and second-generation EGFR-TKIs after 1–2 years. The mechanism of drug resistance for nearly half of cases relates to the T790M mutation. Third-generation EGFR-TKIs that target EGFR-TKI-sensitive mutations and the T790M mutation have been developed [10].

Unfortunately, drug resistance caused by less-common mutations in the EGFR gene and components of signal transduction pathways continues to emerge. In addition to common secondary (T790M) and tertiary (C797S) mutations, other EGFR mutations (such as the L718Q, L796S, and L792H mutations and the exon 20 insertion), MET amplification, phosphatidylinositol 4,5-bisphosphate 3-kinase catalytic subunit alpha (PIK3CA) mutations, HER2 amplification, oncogene fusions, and alterations in cell cycle-related genes have been observed [11] (Fig. 1C). There is an urgent need for better strategies to combat the inevitable molecular-targeted drug resistance associated with third-generation inhibitors. This review aims to provide a comprehensive overview of the mechanisms of resistance to third-generation EGFR-TKIs and to explore new insights and strategies for overcoming acquired resistance.

## Third-generation EGFR-TKIs and drug resistance mechanisms

### The development of third-generation EGFR-TKIs

**Fig. 2:**
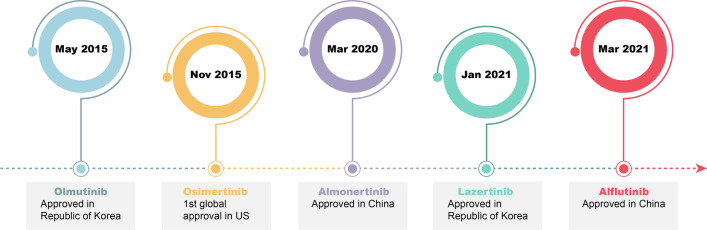
Development of third-generation EGFR-TKIs

The first-generation EGFR-TKIs form hydrogen bonds with Met793 in the ATP binding pocket of EGFR and reversibly compete with ATP for binding. Drug resistance occurs due to the EGFR T790M mutation (Thr790 in the hydrophobic ATP binding site encoded on exon 20 is replaced by methionine), subclonal selection (of a genetically resistant clone), and rare EGFR mutations (such as G719X, S768I, and L861Q). Thereafter, the development of second-generation EGFR-TKIs was reported; these inhibitors have the same quinazoline scaffold as first-generation EGFR-TKIs, but the side chain can irreversibly bind to Cys797 to inhibit the tyrosine kinase activity of EGFR. For example, the anilinoquinazoline derivative forms hydrogen bonds with the backbone of Met793 in the hinge region and interacts with the hydrophobic region. The acrylamide group binds covalently to Cys797 in the active conformation of EGFR, the furanyl group is exposed to solvent, and the 3-chloro-4-fluorophenyl group is situated next to the gatekeeper residue [12–14]. However, mutations such as T790M still emerge upon treatment with second-generation EGFR-TKIs, which have limited selectivity against WT-EGFR, resulting in serious side effects [15]. Fortunately, third-generation covalent inhibitors that bind irreversibly to the target and are mutation-selective have been developed. These compounds were designed based on a new aminopyrimidine scaffold and show preferable biological activities [16]. Early clinical trials have proven that these third-generation EGFR-TKIs are effective in patients with double-mutated tumors (EGFR L858R/T790M or ex19del/T790M) and have high selectivity for mutant EGFR, thereby eliminating the side effects in the skin and gastrointestinal system associated with the nonselective inhibition of WT-EGFR [17]. For example, the crystal structures of rociletinib (CO-1686) in complex with EGFR T790M and EGFR L858R have been published; in EGFR T790M, the anilinopyrimidine group of rociletinib forms hydrogen bonds with the Met793 amide and the carbonyl backbone, whereas in EGFR L858R, hydrophobic interactions between rociletinib and the protein were due to hydrogen bonds between nitrogens in the pyrimidine group and between the fluoromethyl and Thr790. In addition, the acrylamide group in rociletinib covalently binds to Cys797 in the DFG-in/αC-in active conformations [18]. The specificity for EGFR T790M may stem from hydrophobic interactions between the large methionine in mutant EGFR and pyrimidines. Drugs that have been approved for marketing include osimertinib (US), almonertinib (China), lazertinib (South Korea), and alflutinib (China) (Fig. 2).

### Mechanisms of resistance to third-generation EGFR-TKIs

**Fig. 3:**
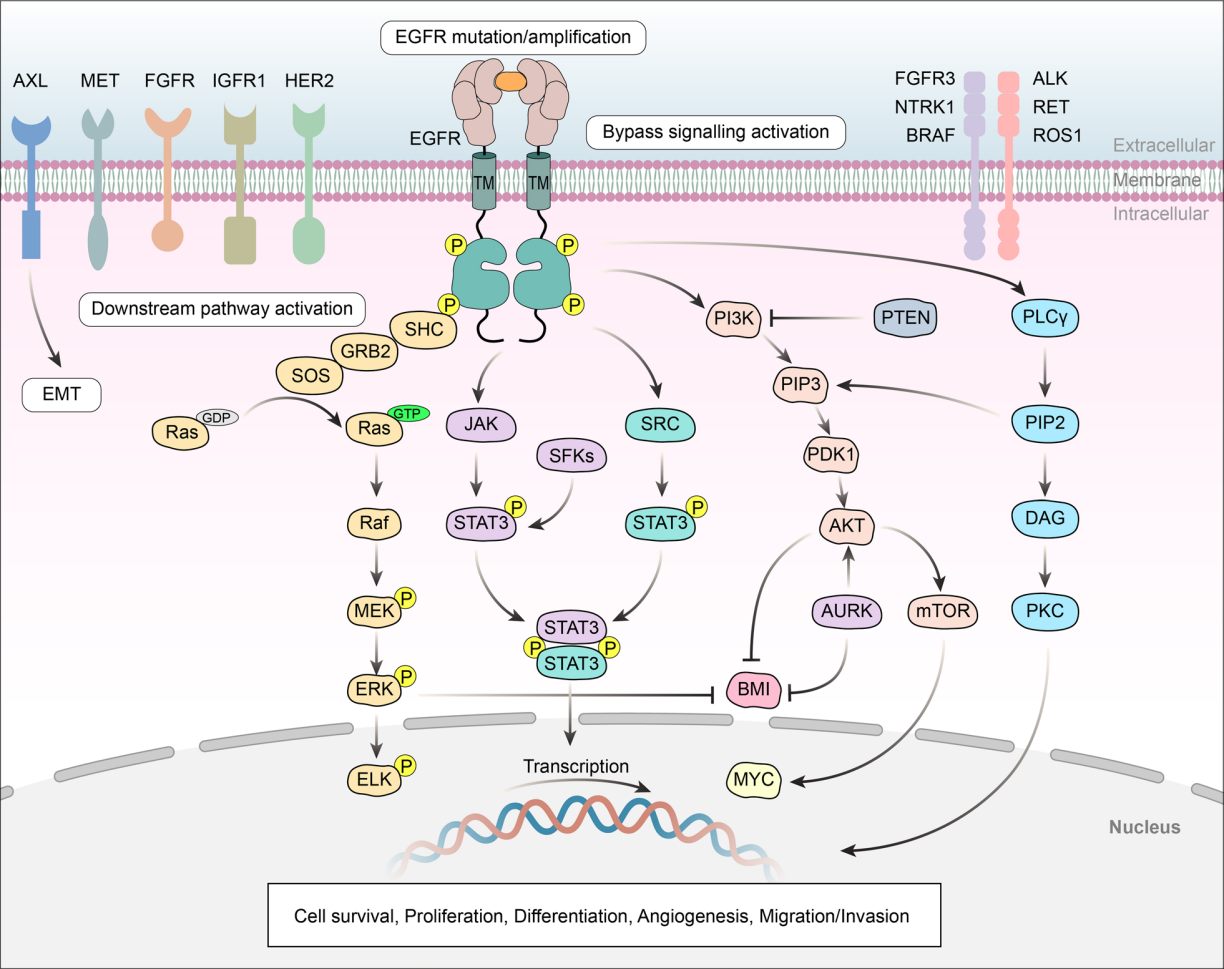
Molecular mechanisms of acquired resistance. The mechanisms include target gene modification, parallel alternative pathway activation, downstream pathway activation, and histological/phenotypic transformation. Both amplification and mutation of receptor tyrosine kinases (RTKs) can induce downstream survival signaling pathways. Moreover, direct overexpression and/or mutation of components of downstream pathways can contribute to acquired resistance by promoting cancer cell survival

Due to the covalent bond between the acrylamide (Michael acceptor) of third-generation EGFR-TKIs and the active thiol in the EGFR kinase domain, highly selective inhibitory activity has been achieved by targeting Cys797 and irreversible binding EGFR; thus, these compounds show excellent antitumor activity. Targeted therapy for patients with EGFR T790M and EGFR-activating mutations showed good efficacy in both first- and second-line settings. In patients who developed resistance to third-generation EGFR-TKIs as first-line therapy, genetic changes such as MET amplification, EGFR C797X mutation, PIK3CA amplification and mutation, HER2 amplification and mutation, K-RAS mutation, and BRAF mutation, as well as changes in cell cycle-related genes and oncogene fusions, have been reported, but no T790M mutations have been detected. The mechanism of resistance to second-line therapy is more complicated. Acquisition or deletion of the T790M mutation has been detected in patients [19], and other EGFR mutations (such as L718Q, L796S, L792H, and exon 20 insertion) have also been observed (Fig. 1B). In addition, the mechanisms of acquired resistance to third-generation EGFR-TKIs include alternative pathway activation and histologic and phenotypic transformation (Fig. 3); the details will be discussed in the following sections.

### Primary/intrinsic resistance

The differential sensitivity of TKIs to different EGFR mutations is a cause of primary drug resistance. In NSCLC patients, the in-frame deletion of exon 19 (ex19del) and the L858R point mutation in exon 21 are the most common somatic mutations, occurring in approximately 80% of cases. During EGFR-TKI treatment, patients with longer median survival have presented with more than 20 unique deletions of exon 19. Intrinsic drug resistance can all be triggered by other nonclassical sensitizing mutations (mainly exon 20 insertion) and inherent secondary genetic changes. Drug-resistant clones (for example, T790M) may already exist within the cancer cell population, leading to drug resistance during treatment [20]. Some studies have found that in nearly 1% of lung cancer patients, 2–3 simultaneous driver mutations can be detected before treatment. Some molecular and genetic changes have been reported to relate to intrinsic drug resistance, such as the lack of K-RAS/phosphatase and tensin homolog (PTEN) expression. These preexisting molecular and genetic alterations can stimulate the Ras/Raf/MEK/ERK and PI3K/AKT downstream pathways to promote cancer progression [21].

### BIM deletion polymorphism

BIM is a proapoptotic member of the B-cell lymphoma-2 (Bcl-2) family [22]. Recent studies showed that lung cancer cells with the BIM deletion polymorphism and EGFR mutation are resistant to third-generation EGFR-TKIs, suggesting that the BIM deletion polymorphism has potential as a biomarker to predict the efficacy of third-generation EGFR-TKIs in patients [22].

### EGFR exon 20 insertion

The molecular mechanism of drug resistance caused by the exon 20 insertion is not fully understood. Eck et al. [23] hypothesized that this mutation prevents binding to EGFR-TKIs due to the addition of residues to the N-lobe of EGFR. The crystal structure of EGFR exon 20 with the D770_N771insNPG insertion shows an unchanged ATP binding pocket and a rigid active conformation, leading to steric hindrance of the drug binding pocket and resistance to EGFR-TKIs.

### Acquired resistance

Acquired drug resistance refers to the process by which tumor cells with prior sensitivity to treatment circumvent the inhibitory effects of drugs by changing their metabolic pathways. The mechanisms of acquired resistance to third-generation EGFR-TKIs can be divided into EGFR-dependent resistance and EGFR-independent resistance [24].

## EGFR-dependent drug resistance mechanisms

### Reappearance of an EGFR mutation

#### C797S mutation

One point mutation of EGFR (C797S) involves the replacement of Cys797 within the ATP binding site (exon 20) with serine [25]. Osimertinib binds covalently and irreversibly to EGFR T790M by interacting with Cys797. When the C797S mutation occurs, the osimertinib binding efficiency decreases [10], resulting in tumor resistance to all third-generation EGFR-TKIs.

#### G796R/D mutation

The G796R mutation has been detected in cancer patients who received treatment with a third-generation EGFR-TKI. Molecular docking predictions revealed that G796R sterically hinders the covalent binding of osimertinib. Because the bulky side chain and hydrophilic group hinder the binding of osimertinib to the hydrophobic region, the change in binding energy renders binding unfavorable. Compared with samples containing the double-mutant EGFR L858R/T790M, those harboring the triple-mutant EGFR L858R/T790M/G796R are 110 times more resistant to osimertinib [26]. G796D was reported for the first time in osimertinib-resistant NSCLC patients. In vitro studies have shown that the G796D mutation causes a 50-fold increase in the growth inhibitory 50% (GI_50_) value of osimertinib. Structural modeling showed that the side chain of the mutated G796D residue collides with the surface of osimertinib, resulting in steric hindrance and energy repulsion and ultimately the loss of binding affinity [27].

#### L792 mutation

The mutations at Leu792 include L792F, L792Y, and L792H. Structural prediction revealed that these mutations introduce a benzene ring or imidazole ring to the side chain of the residue at 792, which spatially disrupts the orientation of osimertinib, thereby potentially affecting the binding of osimertinib to the EGFR ATP binding site [28].

#### M766Q mutation

The homology simulation with the T790M and M766Q double mutant showed that M766Q seems to position T790M in the inhibitor binding site, thereby weakening osimertinib binding [29].

#### Mutations in exon 18

##### EGFR L718Q/V

EGFR L718Q was reported for the first time in a cell model of resistance to third-generation EGFR-TKIs. Subsequent studies have shown that NSCLC with EGFR L858R/T790M/L718Q is resistant to all EGFR-TKIs, but that with only L858R/L718Q remains sensitive to afatinib [30]. The crystallographic model revealed that the L718Q mutation reduces the efficiency of the formation of covalent bonds between the acrylamide warhead and the Cys797 thiol group, thus interfering with the irreversible binding of osimertinib [31, 32]. In addition, L718V resistance mutations in the kinase domain of EGFR have been detected, and these may interfere with the binding of osimertinib to the kinase domain [33]. Of note, EGFR L718Q/V is still sensitive to afatinib [32].

##### EGFR G724S

The G724S mutation in the ATP binding loop enriches this loop in glycine, which can lead to the development of resistance to EGFR-TKIs by changing the protein structure, enhancing ATP affinity, and stabilizing activating mutations [34]. However, this mutation does not lead to resistance to second-generation EGFR inhibitors [34].

#### Compound mutations

A compound mutation refers to the simultaneous detection of two or more different types of EGFR mutations in patient cancer cells [35]. The impact of compound mutations on EGFR-TKI sensitivity is listed in descending order: double classic mutations, compound mutations involving classic mutations and rare mutations, and compound mutations of only rare mutations [36, 37]. These EGFR mutations caused by treatment with third-generation EGFR-TKIs confer resistance to irreversible pyrimidine TKIs but not to quinazoline EGFR inhibitors [38].

### T790M reduction or deletion

Deletion of T790M may result from third-generation EGFR-TKI treatment or may be one of the reasons for drug resistance related to tumor heterogeneity. In patients with EGFR T790M, resistance mechanisms are often associated with the C797S mutation or aberrant activation of compensatory pathways, whereas patients with the deletion of T790M typically exhibit different resistance mechanisms, most of which are not associated with EGFR signaling pathways [39].

### EGFR amplification

Piotrowska and colleagues reported EGFR T790M allele amplification in rociletinib-resistant clones [40]. Nukaga et al. found that amplification of the WT allele of EGFR is sufficient to mediate resistance to third-generation TKIs. The mechanism of drug resistance may be that EGFR gene amplification leads to a relatively low TKI concentration that is insufficient to exert inhibitory activity [41].

### EGFR-independent resistance mechanisms

Not all patients develop resistance to TKIs through EGFR mutation; other pathways of acquiring resistance to third-generation EGFR-TKIs include the activation of alternative or downstream signaling pathways, epithelial interstitial resistance, epithelial–mesenchymal transition (EMT), histologic and phenotypic transformation, oncogene fusion, and cell cycle-related gene abnormalities.

### Bypass signal pathway activation

#### Abnormal activation of MET

There are two main drug resistance mechanisms caused by the abnormal activation of MET: the MET exon 14 skipping mutation (METex14) and MET amplification. METex14 leads to the loss of ubiquitin ligase binding sites, a reduction in receptor ubiquitination, and persistent MET activation, resulting in tumor cell survival and acquired resistance [42]. After treatment with third-generation EGFR-TKIs, MET gene amplification can promote drug resistance by activating MAPK/ERK, which is independent of EGFR [43].

#### HER2 amplification

Hus et al. found that H1975 cells expressing HER2D16 were resistant to osimertinib in vitro. HER2D16 can form a heterodimer with EGFR or a disulfide homodimer, which activates downstream signaling to achieve resistance to osimertinib [44]. HER2D16-driven drug resistance occurs in a manner unrelated to the kinase Src. In addition, other mutations in exon 20 of HER2 have been reported, including point mutations (such as G776C and L755S) and insertions that cause downstream activation [45, 46]. HER2 mutation occurs in approximately 2–4% of NSCLC cases, mostly in lung adenocarcinoma (LUAD) [47]. In NSCLC, HER2 oncogenic amplification occurs in approximately 3% of cases without EGFR-TKI treatment and accounts for approximately 10% of cases with EGFR-TKI resistance [48].

#### AXL activation

AXL is an RTK that regulates cell survival, proliferation, metastasis, and other cellular functions. Abnormalities in the AXL gene can generate acquired resistance to TKIs by activating relevant downstream signaling pathways. Osimertinib was found to trigger AXL activation by closing the negative feedback loop with SPRY4, thus triggering inherent osimertinib resistance [49].

#### Overexpression of HGF

Hepatocyte growth factor (HGF) is the ligand of the proto-oncogene c-Met; it can trigger MET activation through EGFR bypass signaling and induce lung cancer resistance to EGFR-TKIs. Yano et al. [50] found that high expression of HGF was related to the acquired and intrinsic drug resistance to EGFR-TKIs in patients with lung cancer. Tumor specimens from patients with acquired drug resistance showed high expression of HGF in the context of MET amplification and the T790M mutation.

#### Fibroblast growth factor receptor (FGFR) signaling

FGFR is a transmembrane RTK. Studies have shown that FGFR1 is amplified and fibroblast growth factor 2 (FGF2) mRNA levels are increased in patients with osimertinib resistance, suggesting that the FGFR2-FGFR1 autocrine loop may be related to drug resistance [51]. Patients with the T790M mutation have been reported to show disease progression after treatment with osimertinib and nilotinib. The FGFR3-TACC3 fusion was detected in ctDNA [52, 53]. These findings suggest that abnormalities in the FGFR signaling pathway may underlie the mechanism of acquired resistance to third-generation EGFR-TKIs.

#### Insulin-like growth factor receptor 1 (IGF1R)

IGF1R, a transmembrane heterotetrameric protein encoded by the gene located on chromosome 15q26.3, is involved in promoting the growth of tumor cells. Abnormal activation of IGF1R leads to EGFR-TKI resistance [54].

#### Aurora kinases (AURKs)

AURKs are an important category of enzymes within the serine/threonine kinase family consisting of three mammalian isoforms: Aurora kinase A (AURK A), AURK B, and AURK C [55, 56]. AURK A and AURK B are highly expressed in dividing cells and play important roles in mitotic progression. Mammalian AURK A and AURK B share approximately 71% similarity in the carboxy-terminal catalytic domain [57]. Aberrant expression of AURK A and AURK B is involved in a broad range of solid cancers and is associated with adverse prognosis and drug resistance [58, 59]. In addition, Tanaka et al. [60] reported that targeting AURK B can prevent and overcome resistance to EGFR inhibitors in lung cancer by enhancing BIM- and PUMA-mediated apoptosis.

### Downstream signaling pathway activation

The activation of signaling pathways downstream of oncogenic receptors can regulate cell proliferation, cell cycle progression, and cell survival. Therefore, the direct regulation of downstream signaling pathway-related factors can lead to acquired resistance.

#### K-RAS mutation

An epidemiological meta-analysis found that K-RAS mutations are present in NSCLC patients, and all patients with K-RAS mutations were resistant to EGFR-TKIs [61]. K-RAS mutation is related to activation of the RAS-MAPK pathway. The common K-RAS mutations include G12S, G12D, G12A, Q61H, and A146T. Studies have found that inhibiting mutant K-RAS can reduce tumor growth and render NSCLC patients sensitive to EGFR inhibitors [62].

#### BRAF (v-RAF murine sarcoma viral oncogene homologue B1) mutation

BRAF is a serine/threonine protein kinase that plays a key role in the MAPK/ERK pathway, including in EGFR/RAS/RAF signal transduction. BRAF can regulate cell survival, proliferation, differentiation, and apoptosis, as well as tumor induction. Many BRAF mutations (G469A, V600E, and V599E) have been found in cancer, including lung cancer [63]. Ohashi et al. [64] reported that in patients with lung cancer, BRAF mutations can induce acquired resistance to EGFR-TKIs. Preclinical data showed that the BRAF V600E mutation has a strong association with resistance to the third-generation EGFR-TKI osimertinib in patients with T790M-mutated LUAD.

#### PI3K/AKT/mTOR

PIK3CA is a driver gene of LUAD. Mutation of PIK3CA can promote tumor cell invasion and increase the activity of downstream PI3Ks. Studies have shown that PIK3CA amplification or mutation (including E453K, E545K, and H1047R) may occur in patients with osimertinib resistance [52, 65]. Increased PI3K activity leads to the activation of various downstream kinases, thereby increasing PI3K/AKT/mTOR pathway activity in the absence of coupling to upstream EGFR phosphorylation.

#### STAT3 activation

STAT proteins, especially STAT3, are key downstream signal sensors of EGFR activation. In studies on NSCLC, Zhao et al. [66] discovered the clinical significance of JAK2/STAT3 in angiogenesis. Chaib et al. [67] found that osimertinib treatment activates not only STAT3 but also SrcYAP1 signaling, which may act downstream of IL-6 to promote disease progression.

#### Loss of PTEN

PTEN is a tumor suppressor gene that encodes a protein with lipid phosphatase activity and thus regulates cellular protein phosphatase activity. PTEN has dual antitumor effects and is a key component of many signaling pathways in the body. If mutation or deletion of the PTEN gene or downregulation of PTEN expression can reduce or eliminate its antitumor activity [68], loss of PTEN leads to hyperactivation of the PI3K/AKT signaling pathway and resistance to EGFR-TKIs, including osimertinib.

#### Hyperactivation of activated Cdc42-associated kinase 1 (ACK1)

Hyperphosphorylation of ACK1 and the subsequent activation of antiapoptotic signaling through the AKT pathway are associated with resistance to third-generation EGFR-TKIs [69].

#### c-Myc gene

The c-Myc gene is an important member of the MYC gene family. The c-Myc gene can induce cells to proliferate indefinitely and can promote cell division; these activities are related to the occurrence and development of various types of cancer. Studies have shown that c-Myc levels are substantially elevated in different EGFR-mutant NSCLC cell lines with acquired resistance to the third-generation EGFR-TKI osimertinib compared with the corresponding parental cell lines; moreover, these increased levels cannot be reduced by osimertinib. Consistently, c-Myc levels are elevated in the majority of EGFR-mutant NSCLC tissues from patients who relapsed on EGFR-TKI treatment compared with the corresponding baseline c-Myc levels prior to treatment [70]. These findings indicate that c-Myc mediates the therapeutic efficacy of third-generation EGFR-TKIs and the development of acquired resistance to these TKIs.

### Other mechanisms

#### Epithelial–mesenchymal transition (EMT)

In EMT, cancer cells lose their epithelial properties through the loss of E-cadherin, leading to increased vimentin expression and transformation into a mesenchymal phenotype. A previous study found that osimertinib-resistant H1975 cells have EMT characteristics in the absence of other EGFR mutations [71]. EMT is a coordinated process involving multiple regulatory factors, such as EMT-induced transcription factors (EMT-TFs), noncoding RNAs (ncRNAs), and various extracellular signals. EMT-TFs play an important role in all stages of EMT; the most well-known EMT-TFs are members of the SNAIL, ZEB, and TWIST families. Many studies have shown that SLUG and SNAIL overexpression can induce drug resistance [72].

#### miRNAs and EMT

Long noncoding RNAs (lncRNAs) and microRNAs (miRNAs) play important roles in regulating EMT and TKI resistance. Although most miRNAs have been found to inhibit EMT, some have activity that promotes EMT, including miR-21 and miR-155 [73, 74]. Some miRNAs can promote TKI resistance by activating the PI3K/AKT/mTOR signaling pathway; for example, miR-21 and miR-23a can target PTEN and activate AKT, leading to resistance to EGFR-TKIs [75, 76].

#### Epigenetic alterations

Epigenetic modifications involved in cancer initiation and progression include changes in DNA methylation patterns and histone modifications. Epigenetic changes are common in the development and progression of lung cancer [77]. Studies have shown that epigenetic disorders can make cancer patients susceptible to acquired resistance to EGFR-TKIs [78].

#### Oncogene fusion

The AURA-3 and FLAURA trials showed that oncogene fusion might be one mechanism of osimertinib resistance; the identified fusions included transforming growth factor receptor (TGFR)-transforming acidic coiled-coil protein 3 (TACC3), neurotrophic receptor tyrosine kinase 1 (NTRK1)-thrombopoietin mimetic peptide 3 (TMP3), ERC1-RET, SPTBN1-ALK, coiled-coil domain-containing protein 6 (CCDC6)-RET, GOPC-ROS1, AGK-BRAF, NCOA4-RET, ESYT2-BRAF, and echinoderm microtubule-associated protein-like 4 (EML4)-ALK. Oncogene fusions can coexist with the EGFR C797S mutation, MET amplification, and BRAF mutation [79].

#### Cell cycle-related gene abnormalities

Recent studies have shown that changes in cell cycle-related genes, including the CDKN2A E27fs mutation, cyclin D (CCND) amplification, cyclin-dependent kinase 4/6 (CDK4/6) amplification, and cyclin E1 (CCNE1) amplification, can cause resistance to third-generation EGFR-TKIs [65].

#### Histologic and phenotypic transformation

Histopathological transformation to small cell lung cancer (SCLC) from NSCLC has been reported as a mechanism of acquired resistance to EGFR-TKIs in 3–15% of patients [80–83]. Transformed SCLC mainly occurs in Asian patients with adenocarcinoma harboring EGFR-TKI-sensitive mutations (such as the EGFR ex19del/T790M mutation) who are nonsmokers. The widely accepted hypothesis for this transformation posits that adenocarcinoma and SCLC originate from type II alveolar cells. RB1 and TP53 mutations might be involved in SCLC transformation but are not sufficient for the induction of complete transformation. Additional genomic alterations, including those that activate the PI3K/AKT family and downregulate NOTCH signaling and those affecting the MYC and SOX families, AKT pathway activation and other molecules, also participate in the transformation from EGFR-mutant NSCLC. However, the precise mechanisms in other cases are unclear [84]. In addition, squamous cell transformation was recently identified as a mechanism of acquired EGFR-TKI resistance that occurs in approximately 15% of patients who received osimertinib as both first- and second-line therapy. Similar to the case in SCLC transformation, the primary EGFR mutation is preserved in squamous cell transformation [85].

#### Immune escape

EGFR is expressed in different hematopoietic cell types, including macrophages, monocytes, and certain T-cell subsets. Therefore, it is likely that EGFR inhibitors can interfere with the function of these leukocytes. Immune checkpoint inhibitors (ICIs) have adverse effects and poor efficacy in patients with an EGFR mutation or a secondary T790M mutation, largely because of low tumor mutational burden and a noninflamed tumor microenvironment [86–88]. A previous study showed that secreted phosphoprotein 1 (SPP1) promotes macrophage M2 polarization and PD-L1 expression in LUAD, which may influence the response to immunotherapy. SPP1 levels might be a useful marker of immunosuppression in patients with an EGFR mutation and could provide therapeutic insight [89]. In addition, HGF, MET amplification, and EGFR T790M lead to the upregulation of PD-L1 expression in NSCLC and promote immune escape by tumor cells through different mechanisms mediated by the PI3K-Akt, MAPK, and NF-κB pathways [90].

### Strategies for overcoming third-generation EGFR-TKI resistance

#### Fourth-generation EGFR-TKIs: overcoming the L858R/T790M and C797S resistance mutations

Third-generation EGFR-TKIs had the potential for remarkable achievements, if not for the numerous mutations. The C797S mutation, which is a covalent anchor mutation, is located in the ATP binding site of the EGFR tyrosine kinase domain. This missense mutation in exon 20 at position Cys797 blocks the ability of third-generation EGFR-TKIs to form a covalent bond in the ATP binding region, with a consequent decrease in the binding affinity between EGFR and an EGFR-TKI [91]. The combination of the C797S mutation with exon 19 deletion, L858R mutation, or T790M mutation was reported both in vitro and in vivo [91]. Studies have shown that drug-resistant lung cancer cells with two mutations (EGFR-activating mutation/C797S) are sensitive to first- and second-generation EGFR-TKIs. However, lung cancer cells with three mutations (EGFR-activating mutation/T790M/C797S) show resistant to third-generation EGFR-TKIs if the C797S and T790M mutations are both in the trans conformation. Nonetheless, these cells are still sensitive to the combination of first- and third-generation EGFR-TKIs [92]. Of note, if C797S and T790M are mutated in the cis conformation, the cells show resistance to all existing EGFR-TKIs (either alone or in combination) [93]. The resistance to third-generation EGFR-TKIs caused by the trans-C797S mutation can be overcome by drugs targeting different kinase binding sites, including allosteric inhibitors, ATP-competitive inhibitors, and “dual-site” inhibitors that occupy both the ATP binding site and an allosteric site.

#### Allosteric inhibitors

EGFR has three binding sites: an inactive site, a competitive ATP binding site, and an allosteric site. Ligands and drugs cannot bind the inactive site. Recent studies have mostly focused on either ATP-competitive inhibitors targeting the ATP binding site or molecules that bind the allosteric site, which causes a conformational change in the protein that inhibits the signaling cascade [94]. To overcome the resistance of EGFR-TKIs mediated by the T790M and C797S mutations and to further identify and explore compounds that bind outside the ATP binding domain of EGFR, researchers have pursued the development of allosteric inhibitors, and this appears to be a promising strategy. The newly developed fourth-generation mutant-selective allosteric inhibitors can overcome the T790M and C797S mutations that develop in response to third-generation EGFR-TKIs by binding to sites outside the ATP binding pocket of EGFR.

**Fig. 4:**
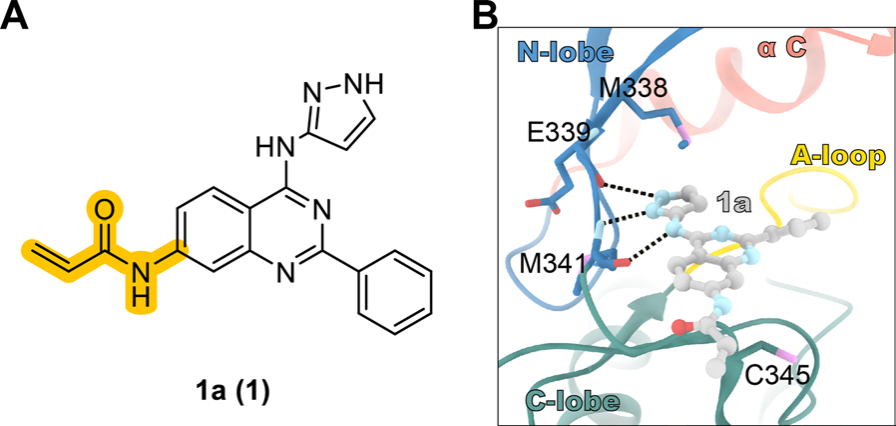
Screening hit **1a (1)** from a phenotypic screen of NSCLC cell lines. **A** Structure of **1a (1)**. **B** X-ray crystal structure of **1a (1)** in complex with c-Src-DM (PDB code: 5D12)

Through molecular phenotypic screening, Engel et al. obtained quinazoline compound 1a **(1)**, which specifically inhibits the drug-resistant H1975 cell line (L858R/T790M); further modification addressed the problem of off-target activity (nonspecific inhibition). X-ray crystallography verified that compound 1a (**1**) fits well in the tyrosine kinase domain of c-Src [95] (Fig. 4).

**Fig. 5:**
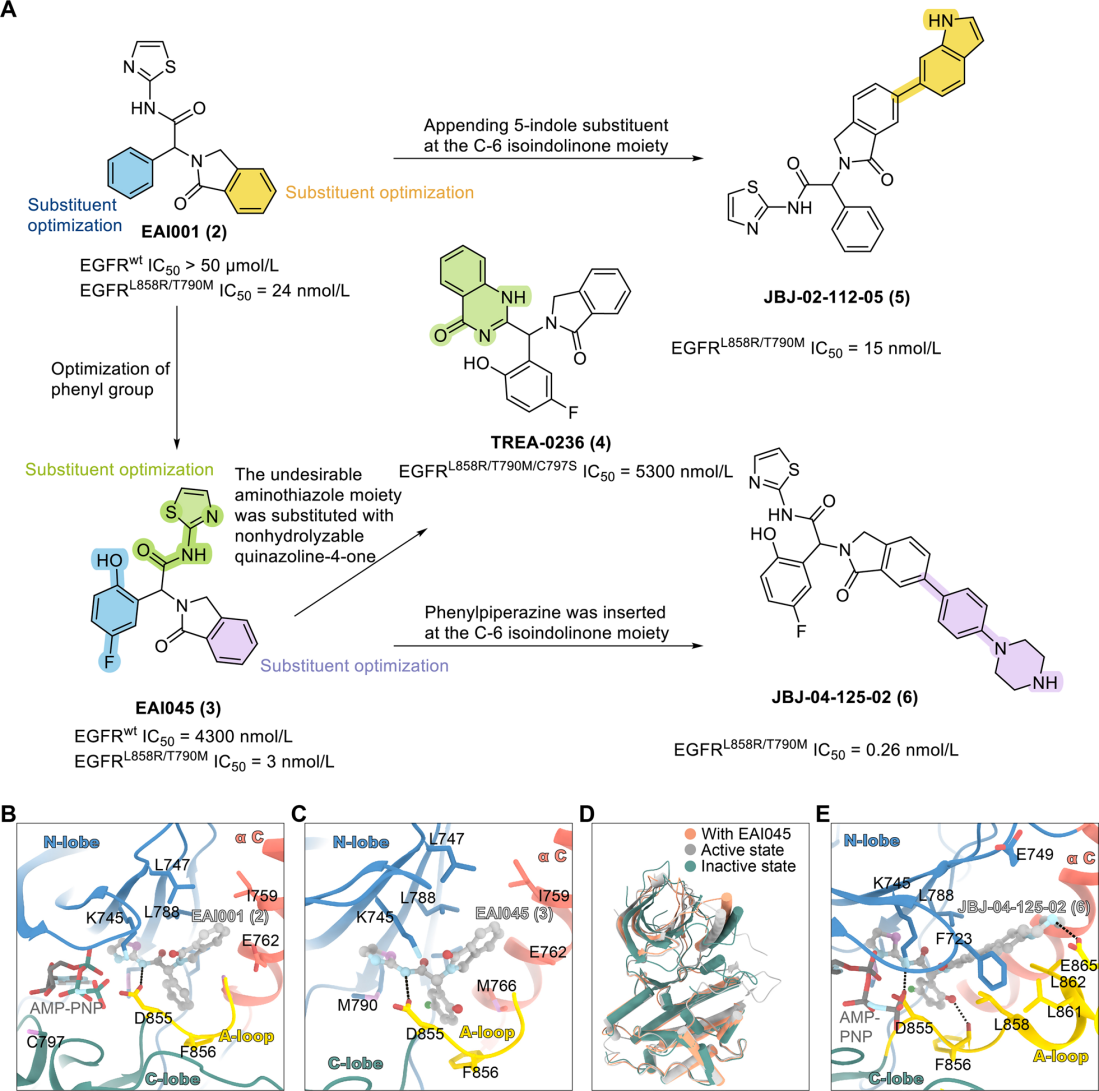
Chemical structures and structure–activity relationships of allosteric inhibitors. **A** The rational design of TERA-0236 (**4**), JBJ-02-112-05 (**5**), and JBJ-04-125-02 (**6**) and their inhibitory activities against EGFR. **B** X-ray cocrystal structure of EGFR with EAI001 (**2**) (PDB code: 5D41). **C** X-ray cocrystal structure of EGFR with EAI045 (**3**). **D** X-ray cocrystal structure of EGFR with EAI045 (in the active and inactive states) (PDB code: 5ZWJ). **E** X-ray cocrystal structure of EGFR with JBJ-04-125-02 (**6**) (PDB code: 6DUK)

Jia et al. conducted counter-screening of active compounds against WT-EGFR and discovered the first non-ATP-competitive allosteric EGFR L858R/T790M/C797S inhibitor based on the thiazolamide scaffold (EAI001, **2**) [96]. The X-ray crystal structure of EAI001 (**2**) in complex with EGFR T790M shows that EAI001 (**2**) can bind to the allosteric site of this receptor in the form of a “three-bladed propeller,” partly due to the outward displacement of the C-helix in the inactive conformation of the kinase. The hydrophilic side chain of the WT gatekeeper residue (Thr) cannot adapt to the thiazole of EAI001; therefore, there is no favorable interaction. The thiazole of EAI001 closely interacts with the hydrophobic side chain of Met790; specifically, aminothiazole group of EAI001 directly binds to Met790. The carbonyl oxygen of the isoindoline-1-one moiety is inserted between the mutant gatekeeper residue (Met) and the active site residue Lys745, forming another hydrogen bond with the ε-amine of the Lys745 side chain. The NH group of formamide acts as a hydrogen bond donor for Asp855 in the DFG motif. The cationic phenyl group occupies the hydrophobic pocket formed by Met766, Leu777, and Phe856. The 1-oxindolinyl group is exposed along the C-helix and extends to the solvent-accessible area. The ATP analog adenylyl imidodiphosphate (AMP-PNP) binds the active site cavity in an expected manner. The half maximal inhibitory concentration (IC_50_) of EAI001 (**2**) for EGFR L858R/T790M is 24 nmol/L, which is lower than that for WT-EGFR (IC_50_ > 50 μmol/L). The IC_50_s of EAI001 (**2**) for EGFR L858R and EGFR T790M is 0.75 μmol/L and 1.7 μmol/L, respectively. By introducing ortho-hydroxyl and meta-fluorine atoms on the benzene ring of EAI001 (**2**), the researchers synthesized another compound, EAI045 (**3**), that binds more tightly than EAI001 (**2**) to EGFR [96]. However, EAI045 has a major drawback: it must be used in combination with cetuximab to preserve its efficacy. While EAI045 (**3**) has good selectivity for WT-EGFR, cetuximab is expected to have off-target effects in clinical use. Lee et al. [97] designed the EGFR allosteric inhibitor TREA-0236 (**4**) based on the structure–activity relationships of EAI045 (**3**). The structure of EAI045 (**3**) was modified by cyclization, wherein the 2-aminothiazole amide was converted to quinazoline-4-one. To minimize hematological and methemoglobinemia toxicity and to obtain better safety and pharmacokinetic parameters, To et al. linked the 5-indole substituent to the isoindolinone of EAI001 (**2**) and obtained a new EGFR allosteric compound, JBJ-02-112-05 (**5**), with an IC_50_ of 15 nmol/L for EGFR L858R/T790M [98]. Additionally, EAI045 (**3**) was further optimized to generate another EGFR allosteric inhibitor, JBJ-04-125-02 (**6**), in which the 2-hydroxy-5-fluorophenyl of EAI045 (**3**) was combined with the phenylpiperazine on isoindolinone. This compound showed a significantly increased ability to inhibit EGFR L858R/T790M, with an IC_50_ of 0.26 nmol/L. Interestingly, combination with osimertinib enhanced the efficacy of JBJ-04-125-02 (**6**) and improved the targeting of JBJ-04-125-02 (**6**) to cancer cells [98], indicating that the combined use of covalent mutant-selective ATP-competitive inhibitors and EGFR allosteric inhibitors may be an effective treatment strategy for patients with EGFR-mutant disease (Fig. 5). Encouraged by the advantages of inhibiting allosteric sites in the EGFR tyrosine kinase domain, researchers have extensively designed and optimized allosteric inhibitors for EGFR [98–101], as shown in Table 1.

**Table 1:** EGFR allosteric inhibitors

Compound (reference)	Structure	Activity	Interaction with EGFR allosteric site
**7, DDC4002** [100]		EGFR^L858R/T790M^ IC_50_ = 10 nmol/L EGFR^L858R/T790M/C797S^ IC_50_ = 59 nmol/L	Phe856
**8** [100]		EGFR^L858R/T790M/C797S^ IC_50_ = 19 nmol/L	Phe856
**9** [100]		EGFR^L858R/T790M/C797S^ IC_50_ = 23 nmol/L	Phe856
**10** [100]		EGFR^L858R/T790M/C797S^ IC_50_ = 13 nmol/L	Phe856
**11** [100, 101]		EGFR^L858R/T790M^ IC_50_ = 1 nmol/L EGFR^L858R/T790M/C797S^ IC_50_ = 5 nmol/L	Phe856
**12** [100, 101]		EGFR^L858R/T790M^ IC_50_ = 3 nmol/L EGFR^L858R/T790M/C797S^ IC_50_ = 4 nmol/L	Phe856

The novelty of allosteric sites has attracted the attention of researchers, and these sites have become the most promising targets for the development of drugs for NSCLC and other diseases. Fourth-generation EGFR-TKIs require further investigation and development so that they are suitable as single-agent drugs targeting EGFR ex19del/T790M/C797S [98]. Allosteric inhibitors have now entered the stage of rapid development and are expected to enter clinical trials soon, with the goal of benefitting more patients.

#### ATP-competitive inhibitors

ATP-competitive inhibitors form one to three hydrogen bonds with amino acids in the hinge region of the target kinase, thereby mimicking the characteristic hydrogen bonds formed by the adenine ring of ATP. This type of inhibitor usually consists of a heterocyclic ring system that occupies the purine binding site, where it acts as a side chain scaffold that occupies the adjacent hydrophobic regions I and II. A high physiological or intracellular concentration of ATP may block the phosphotransferase activity of the target. The size of the amino acid side chain at the gatekeeper residue determines the relative accessibility of the hydrophobic pocket near the ATP binding site. To overcome drug resistance related to triple-mutant EGFR, it is particularly crucial to develop new ATP-competitive inhibitors based on structural design and optimization. Many ATP-competitive inhibitors have been reported; below, we summarize recent ATP-competitive inhibitors that can overcome the resistance to third-generation EGFR inhibitors (Table 2) [102–119].

**Table 2:** ATP-competitive EGFR inhibitors

Compound (reference)	Structure	Enzymatic activity	Biological activity	DMPK profile
**13, JND3229** [102]		EGFR^L858R/T790M/C797S^ IC_50_ = 5.8 nmol/L	BaF3-EGFR^L858R/T790M/C797S^ IC_50_ = 510 nmol/L BaF3-EGFR^Del19/T790M/C797S^ IC_50_ = 320 nmol/L	N/A*
**14** [103]		EGFR^WT^ IC_50_ = 16 nmol/L EGFR^L858R/T790M/C797S^ IC_50_ = 88 nmol/L	A431 GI_50_ = 3600 nmol/L H1975 GI_50_ = 140 nmol/L	N/A
**15** [104]		EGFR^L858R/T790M/C797S^ IC_50_ = 8 nmol/L	A431 EC_50_ = 4000 nmol/L H1975 EC_50_ = 400 nmol/L	N/A
**16** [105, 106]		EGFR^L858R/T790M/C797S^ IC_50_ = 630 nmol/L	H1975 IC_50_ = 1200 nmol/L	Liver microsomes (Human): *t*_1/2_ (min) = 66.6, CLint (mL/min/kg) = 20.8
**17** [107]		EGFR^WT^ IC_50_ > 1000 nmol/L EGFR^L858R/T790M/C797S^ IC_50_ = 27.5 nmol/L	BaF3-EGFR^L858R/T790M/C797S^ IC_50_ = 662 nmol/L	*T*_1/2_ (rat, minutes) = 8.36, CLint (mL/min/kg) = 297.12
**18** [108]		EGFR^L858R/T790M/C797S^ IC_50_ = 7.2 nmol/L	HCC827 IC_50_ = 44 nmol/L H1975 IC_50_ = 400 nmol/L	N/A
**19** [109, 110]		EGFR^L858R/T790M/C797S^ IC_50_ = 18 nmol/L	HCC827 IC_50_ = 0.88 nmol/L H1975 IC_50_ = 200 nmol/L A549 IC_50_ = 2910 nmol/L A431 IC_50_ > 10,000 nmol/L	N/A
**20** [111]		EGFR^L858R/T790M/C797S^ IC_50_ = 8.5 nmol/L	EGFR CTG EC_50_: HCC827 IC_50_ < 14 nmol/L H1975 IC_50_ = 51 ± 19 nmol/L A431 IC_50_ = 1675 ± 402 nmol/L A549 IC_50_ = 3700 nmol/L H358 IC_50_ = 3700 nmol/L	In vivo PK (mice, IP, 20 mg/kg): AUC_free_ (h∙ng/mL) = 8.6 *t*_1/2_ (h) = 1.2 *C*_max,free_ (μmol/L) = 0.012
**21** [112]		EGFR^L858R/T790M/C797S^ IC_50_ = 3.1 nmol/L	H1975 IC_50_ = 0.12 ± 0.09 μmol/L BaF3-EGFR^L858R/T790M/C797S^ IC_50_ = 0.29 μmol/L BaF3-EGFR^19D/T790M/C797S^ IC_50_ = 0.31 μmol/L	N/A
**22** [113]		EGFR^WT^ IC_50_ > 1000 nmol/L EGFR^L858R/T790M/C797S^ IC_50_ = 218.3 nmol/L EGFR^Del19/T790M/C797S^ IC_50_ = 15.3 nmol/L	H1975 IC_50_ = 16,180 nmol/L A431 IC_50_ = 20,480 nmol/L BaF3-EGFR^Del19/T790M/C797S^ IC_50_ = 8510 nmol/L	N/A
**23** [114]		EGFR^WT^ IC_50_ = 430 nmol/L EGFR^Del19/T790M/C797S^ IC_50_ = 0.2 nmol/L	BaF3-EGFR^WT^ IC_50_ = 1000 nmol/L BaF3-EGFR^Del19^ IC_50_ = 180 nmol/L BaF3-EGFR^Del19/T790M^ IC_50_ = 99 nmol/L BaF3-EGFR^Del19/T790M/C797S^ IC_50_ = 63 nmol/L	N/A
**24** [115]		N/A	Biochemical potency: BaF3 cells IC_50_ < 100 nmol/L Antiproliferative activity: BaF3 cells IC_50_ < 100 nmol/L	N/A
**25** [116]		N/A	Antiproliferative activity: PC-9-EGFR^L858R/T790M/C797S^ IC_50_ = 595.7 nmol/L PC-9-EGFR^Del19/T790M/C797S^ IC_50_ = 739.9 nmol/L A549 IC_50_ = 2861.7 nmol/L BaF3-EGFR^WT^ IC_50_ = 519.82 nmol/L BaF3-EGFR^L858R/T790M/C797S^ IC_50_ = 0.16 nmol/L BaF3-EGFR^Del19/T790M/C797S^ IC_50_ = 0.23 nmol/L	N/A
**26** [117]		N/A	BaF3-EGFR^WT^ IC_50_ = 540 nmol/L BaF3-EGFR^L858R/T790M/C797S^ IC_50_ = 48.2 nmol/L BaF3-EGFR^Del19/T790M/C797S^ IC_50_ = 12 nmol/L	N/A
**27** [115]		EGFR^WT^ IC_50_ = 7.92 nmol/L EGFR^L858R/T790M/C797S^ IC_50_ = 0.218 nmol/L EGFR^Del19/T790M/C797S^ IC_50_ = 0.16 nmol/L	Antiproliferative Activity: A431 IC_50_ = 154 nmol/L BaF3-EGFR^Del19/T790M/C797S^ IC_50_ = 22 nmol/L	In vivo PK (mice, per os, 15 mg/kg): AUC_0-last_ = 57,037 (nmol/L∙h) *t*_1/2_ (h) = 10.0 Plasma (nmol/L), 2 h = 3553 Tumor (nmol/kg), 2 h = 16,667
**28** [118]		EGFR^WT^ IC_50_ = 3.8 nmol/L EGFR^L858R/T790M/C797S^ IC_50_ = 38.1 nmol/L	BaF3-EGFR^L858R/T790M/C797S^ IC_50_ < 1000 nmol/L	N/A
**29, UPR1444** [119]		EGFR^WT^ IC_50_ = 30 ± 4.8 nmol/L EGFR^L858R/T790M/C797S^ IC_50_ = 110 ± 33 nmol/L	N/A	N/A

*N/A not available

#### “Dual-site” inhibitors: occupying both the ATP binding site and the allosteric site

Based on the non-ATP-competitive EGFR L858R/T790M/C797S inhibitor EAI001 reported by Jia et al., the more potent compound EAI045 (**3**) was obtained through structural optimization [96]. EAI045 (**3**) binds to the allosteric site created by the outward displacement of the αC helix of EGFR, located next to the ATP binding pocket. Facilitated by molecular docking, researchers developed a series of new compounds that noncovalently occupy both the EGFR ATP binding site and the allosteric site; these fourth-generation reversible EGFR inhibitors have improved binding affinity for EGFR L858R/T790M/C797S, effectively compete with ATP, and further overcome resistance to third-generation EGFR inhibitors.

**Fig. 6:**
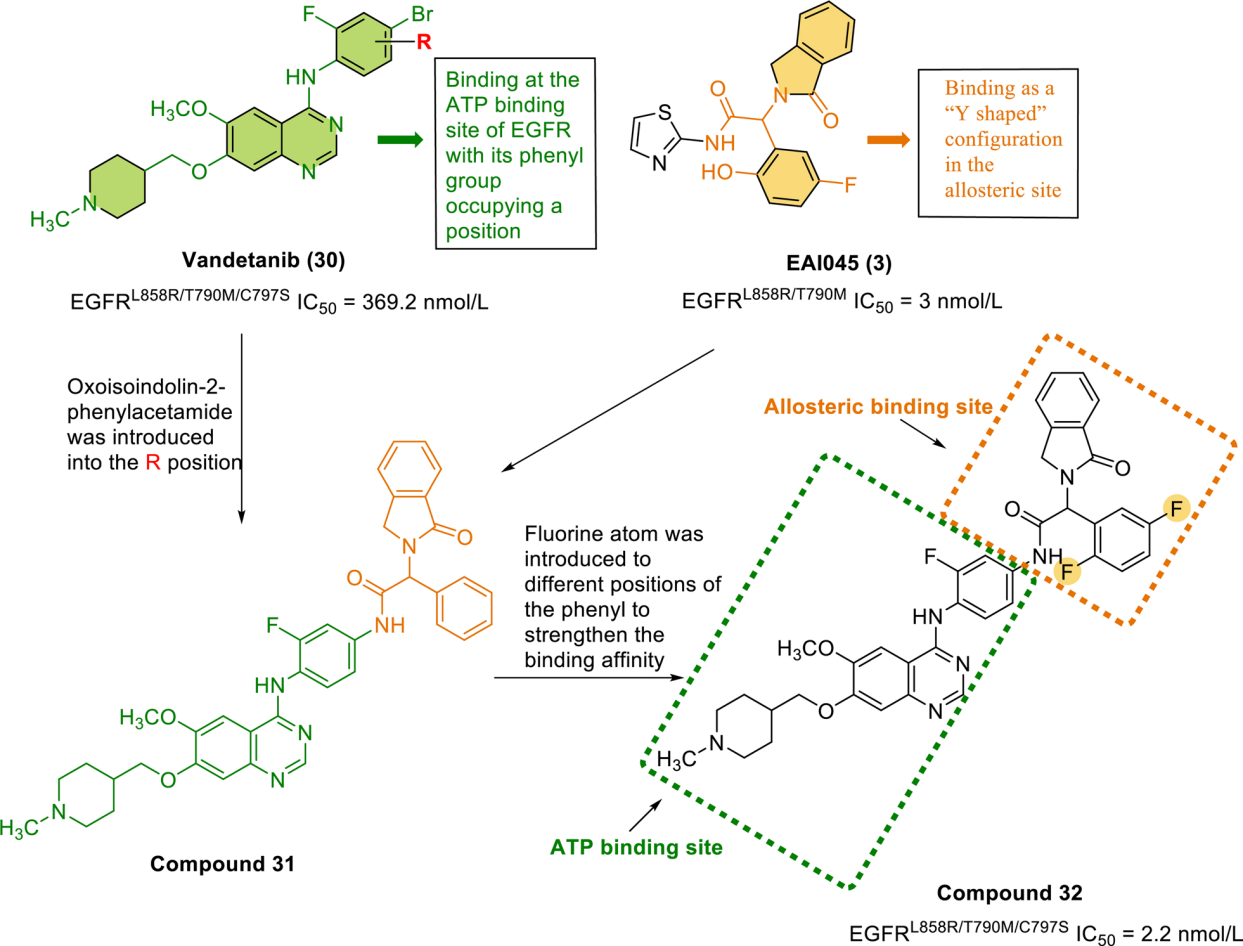
Chemical structures and structure–activity relationships of dual-site inhibitors: the rational design of compound **32** and its inhibitory activity against EGFR

The compound vandetanib (**30**) [120] is a known EGFR inhibitor that shows moderate efficacy against EGFR L858R/T790M/C797S, with an IC_50_ value of 369.2 nmol/L. Via molecular docking simulation, Li et al. found that vandetanib can extend to the EGFR ATP binding pocket (gscore =  − 8.2 kcal/mol). The docking model of vandetanib with EGFR T790M/V948R shows that the phenyl group of vandetanib binds the ATP binding site of EGFR, occupying a position such that it resembles the thiazole moiety of EAI001 (**2**). EAI001 (**2**) binds as a *Y*-shaped constellation in the allosteric site [121]. Modifying vandetanib to occupy both the ATP binding site and the allosteric site may be an effective way to improve its biological activity against EGFR L858R/T790M/C797S. To promote occupation of the allosteric site of EGFR, the structure of EAI045 (**3**) was modified such that the hydrophobic group oxyisoindole-2 phenylacetamide was introduced with an amide bond as the linker, generating compound **31**. With this compound as a new lead, three moieties, namely the allosteric targeting region, the hinge targeting region, and the solvent exposure region, were studied and optimized. Finally, the EGFR L858R/T790M/C797S reversible inhibitor compound **32** (Fig. 6) was obtained, with an IC_50_ value of 2.2 nmol/L. The docking simulation showed that compound **32** occupies both the ATP binding region and the allosteric region. In addition, it extensively interacts with residues in the allosteric region, the solvent-accessible region, and the hinge region. The phenyl of the *Y*-shaped group (oxoisoindolin-2-phenylacetamide) and Phe856 of the allosteric cavity form *π*–*π* stacking interactions. Inside the ATP binding region, hydrogen bonds are formed between the quinazoline ring and the hinge residue Met793. In addition, the piperidine tail is surrounded by the solvent-exposed region. At a concentration of 0.1 μmol/L, compound **32** almost completely inhibited the phosphorylation of EGFR, showing comparable potency to that of EAI045 (**3**).

**Fig. 7:**
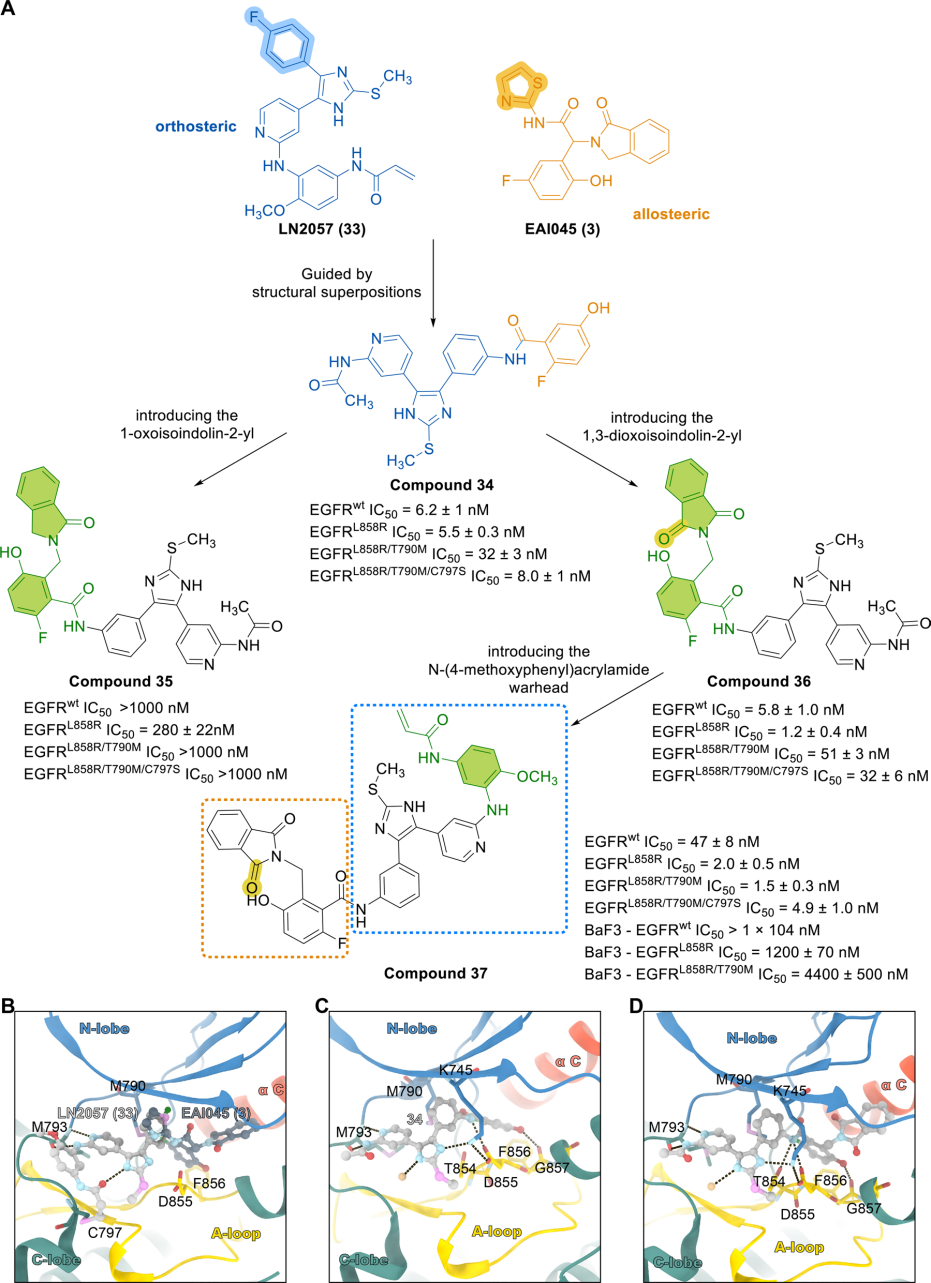
Chemical structures and structure–activity relationships of dual-site inhibitors. **A** Structure-guided design and synthesis of mutant-selective lead compounds and their inhibitory activities against EGFR. **B** Structural superposition of the ATP site binding inhibitor LN2057 (PDB code: 6V6K) and the allosteric inhibitor EAI045 (PDB code: 6P1L); **C** X-ray cocrystal structure of EGFR T790M/V948R with compound **34** (PDB code: 6WA2); **D** X-ray cocrystal structure of EGFR T790M/V948R with compound **36** (PDB code: 6WXN)

To further design more potent inhibitors spanning both binding sites, considering the proximity of the ortho and allosteric positions, Wittlinger F et al. compared the binding of the EGFR ATP site inhibitor LN2057 (**33**) with the allosteric inhibitor EAI045 (**3**) and found that the 4-fluorophenyl of LN2057 (**33**) and the thiazole of EAI045 (**3**) had the same binding position [122]. Based on this, researchers designed and synthesized a series of compounds that combined a large portion of the isomerization inhibitor EAI045 (**3**) with the pyridyl-imidazole skeleton. For compound **34**, the pyridinylimidazole scaffold partly binds the 2-fluoro-5-hydroxyphenyl moiety of EAI045 (**3**); 1-oxoisoindoline-2-yl was introduced into compound **35**; and 1,3-dioxoisoindoline-2-yl was added to generate compound **36** to further explore the structure–activity relationship of the allosteric site. In addition, an *N*-(4-methoxyphenyl)acrylamide warhead was introduced to produce compound **37**, and the influence of the C797-targeting capacity of these chimeric compounds, which are expected to form a covalent bond with C797, was assessed. The X-ray cocrystal analysis of the binding mode with EGFR T790M/V948R (Fig. 7) revealed that compounds **34** and **36** bind in the same way. Taking compound **36** as an example, the aminopyridine moiety forms a hydrogen bond with the M793 residue in the hinge region. The inhibitor is anchored at the ATP binding site, and the N atom of the imidazole moiety forms a hydrogen bond with K745, which is essential for the strong reversible binding of the imidazole skeleton. The phenylamide bond extending into the allosteric pocket is directed toward the T790M mutation, and the N atom on the amide forms hydrogen bonds with the T854 and D855 residues. Despite considerable efforts, the X-ray crystal structure of compound **37** in complex with EGFR was not obtained. Compound **37** was computationally docked to the EGFR T790M/V948R kinase domain, and the result was the same as that for compound **36**. The methoxyphenyl acrylamide formed a covalent bond with C797. Importantly, no covalent binding of compound **37** to the EGFR L858R/T790M/C797S kinase domain was observed, confirming that this compound is a noncovalent inhibitor.

The inhibitory activity of the above compounds was tested, and the results showed that compound **34** exhibited strong inhibitory activity against all mutants, with IC_50_ values of 5–32 nmol/L, indicating that the introduction of 2-fluoro-5-hydroxyphenyl alone did not increase selectivity. With the introduction of oxyisoindolin-2-yl, the inhibitory activity of compound **35** decreased, but a certain inhibitory effect against EGFR L858R was observed. Furthermore, compound **1** inhibited all three EGFR mutants at the low nanomolar range. Compound **37** showed a moderate degree of mutation selectivity for WT-EGFR, possibly due to the methoxyphenyl acrylamide group. To assess the kinase selectivity of compound **37**, a kinome screen including 335 WT kinases was performed; compound **37** exhibited high selectivity, with a selectivity score of 0.006 at an inhibitor concentration of 1 μmol/L. Next, the antiproliferative activity of these compounds was evaluated in Ba/F3 cells stably transfected with WT-EGFR, EGFR L858R, EGFR L858R/T790M, or EGFR L858R/T790M/C797S. Among the compounds, compound **37** showed an antiproliferative effect in the EGFR L858R and EGFR L858R/T790M cell lines, with IC_50_ values in the micromolar range in the presence and absence of cetuximab. The IC_50_ value of compound **37** in EGFR L858R Ba/F3 cells (1.2 ± 0.07 μmol/L) was comparable to that of EAI045 (**3**) combined with cetuximab (840 ± 700 nmol/L). Although compound **37** is potent and selective for kinases, its cellular activity is suboptimal. Kinase selectivity was achieved by increasing the molecular weight of the lead compound and increasing the number of hydrogen bond donors and acceptors, but these changes may have produced limited cell permeability and effects on cell viability; thus, this compound lacked sufficient activity in cells expressing EGFR L858R/T790M/C797S.

The selective EGFR inhibitor (compound **37**) designed and developed in this study can bind to both the ATP site and the allosteric site of the EGFR kinase domain. Adding allosteric inhibitor elements to the compound skeleton at the ATP binding site contributes to the mutation selectivity of these compounds. The designed compound **37** has good kinase activity but nonideal cell activity. Future research and development could optimize the structure of this lead compound to further enhance its cellular activity.

### PROTAC technology

#### Allosteric EGFR degrader

Resistance to third-generation EGFR-TKIs is a major obstacle to clinical targeted therapy. Due to changes in the EGFR protein [123], some kinase inhibitors are restricted to the catalytic pocket [124]. A proteolysis-targeting chimera (PROTAC) induces the proteasomal degradation of the target by recruiting it to a specific E3 ligase. The eradication of EGFR protein from cancer cells provides a promising strategy for overcoming drug resistance. The allosteric EGFR degrader is a heterobifunctional compound based on allosteric EGFR inhibitors. It includes a small molecule (protein-of-interest (POI) ligand) that binds the target protein and a small-molecule E3 ligase ligand that recruits cereblon (CRBN), von Hippel–Lindau (VHL), cellular inhibitor of apoptosis protein 1 (cIAP1) or murine double minute 2 (MDM2). After the addition of a linker connecting the two parts [125, 126], these chimeras can degrade mutant EGFR without affecting WT-EGFR.

Compared with classic “occupying” inhibitors, allosteric EGFR degraders can completely eliminate the function of the target protein, thereby improving the phenotypic potency. Moreover, since PROTAC molecules usually do not require strong binding to targets or long-term retention to achieve protein degradation, the development of drug-induced resistance mutations may be prevented. Compared with kinase inhibitors, PROTACs have the advantages of activity at lower concentrations, limited dose-dependent toxicity, and the potential to overcome drug resistance and target drug refractory disease [127–132]. These molecules have attracted considerable attention from academia and industry and have become an attractive therapeutic strategy in drug discovery.

Based on EAI001 (**2**), a compound that buries deeply in the allosteric pocket [96], Jang et al. introduced 1-(pyridin-2-yl)piperazine at the 6 position of isoindolinone and synthesized JBJ-07-149 (**38**), which has an IC_50_ value of 1.1 nmol/L for EGFR L858R/T790M. In combination with cetuximab, JBJ-07-149 has a half maximal effective concentration (EC_50_) of 0.148 nmol/L for EGFR L858R/T790M. However, this compound was less potent in the proliferation assay (EC_50_ = 4.9 nmol/L) [133].

**Fig. 8:**
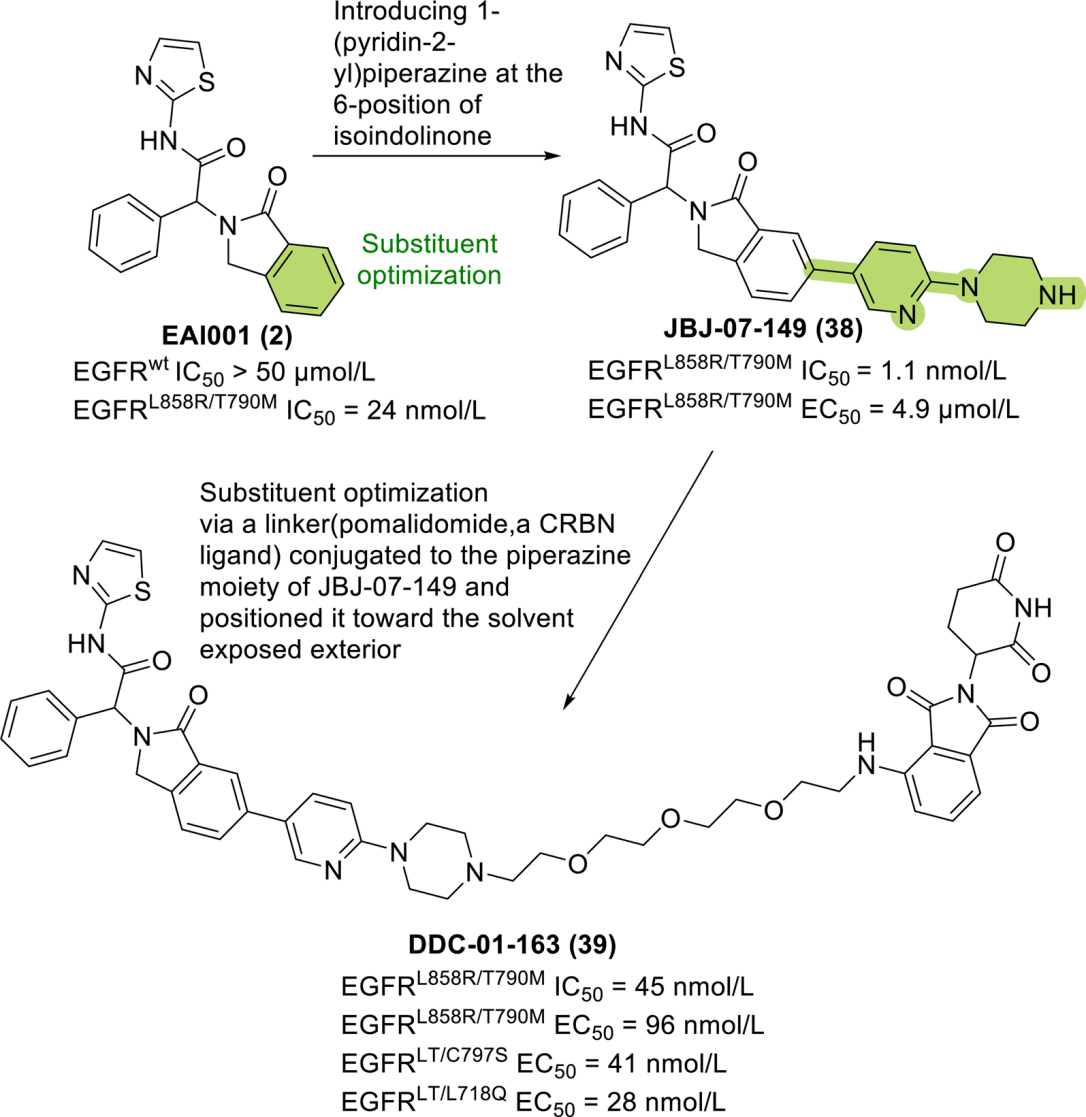
Chemical structures and structure–activity relationships of an EGFR-targeted PROTAC: the rational design of DDC-01-163 (**39**)

Based on JBJ-07-149 (**38**), different linkers that bind the piperazine group and connect the CRBN ligand were evaluated. The compound with 3-PEG as the linker (DDC-01-163, **39**) showed the strongest antiproliferative activity for EGFR L858R/T790M (Fig. 8). DDC-01-163 (**39**) induced the selective degradation mutant EGFR and inhibited the proliferation of cells expressing mutant EGFR in a dose- and time-dependent manner. DDC-01-163 (**39**) showed no activity in WT-EGFR Ba/F3 cells (EC_50_ > 10 μmol/L) but inhibited the proliferation of EGFR L858R/T790M Ba/F3 cells, including those expressing EGFR L858R/T790M (EC_50_ = 0.096 μmol/L), EGFR L858R/T790M/C797S (EC_50_ = 0.041 μmol/L) and EGFR L858R/T790M/L718Q (EC_50_ = 0.028 μmol/L). The results in H1975 cells were consistent with those in Ba/F3 cells. Osimertinib-resistant cell lines treated with 0.1 μmol/L DDC-01-163 (**39**) showed EGFR L858R/T790M/C797S and EGFR L858R/T790M/L718Q degradation rates of 74% and 71%, respectively.

**Fig. 9:**
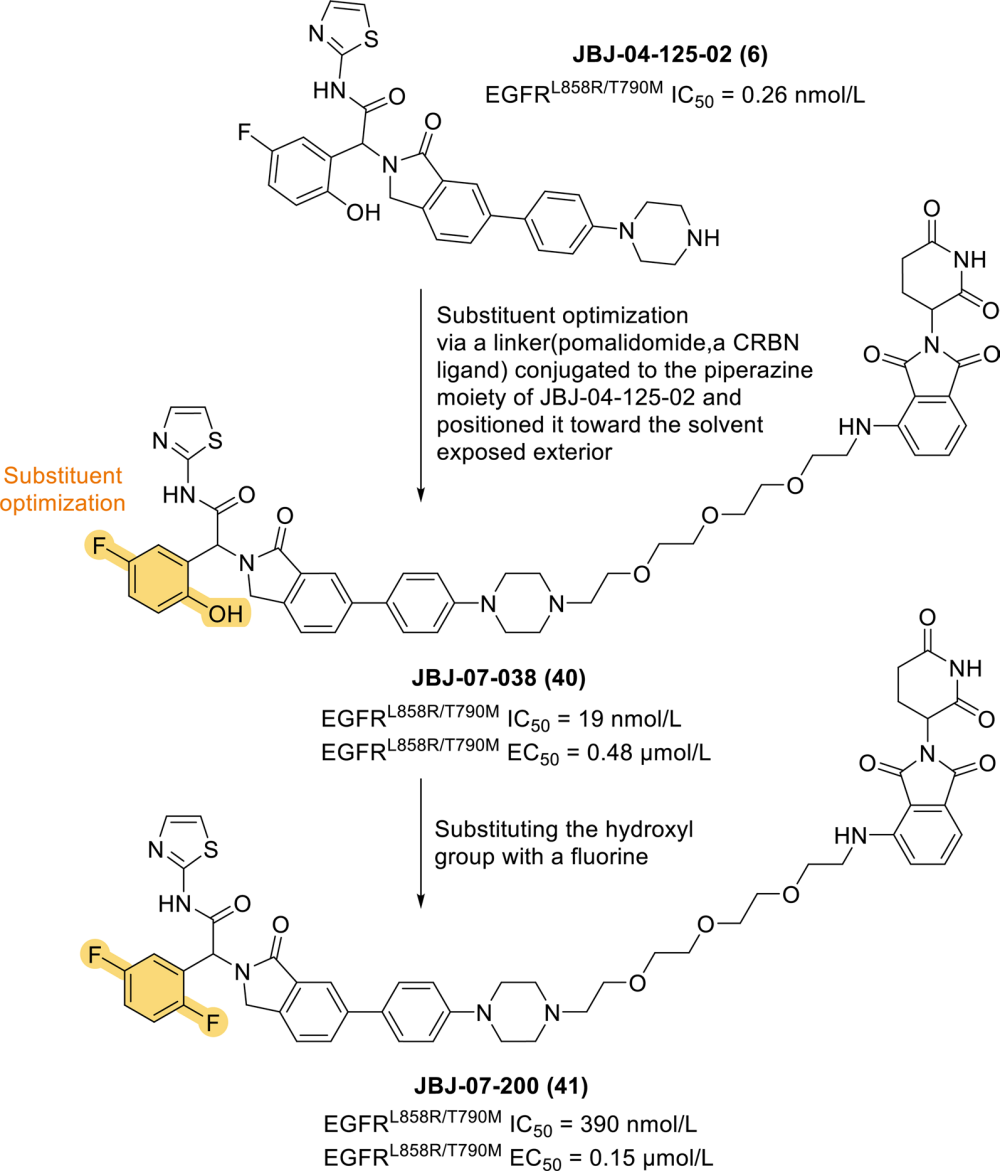
Chemical structures and EGFR inhibitory activities of EGFR-targeted PROTACs: the rational design of JBJ-07-200 (**41**)

Jang et al. also identified the 2-hydroxy-5-fluorophenyl allosteric inhibitor JBJ-04-125-02 (**6**), which can be used as a single agent to inhibit the proliferation of Ba/F3 cells. Following the same strategy as that used to develop DDC-01-163 (**39**), this group designed JBJ-04-125-02 (**6**) as a PROTAC molecule and synthesized the allosteric EGFR degrader JBJ-07–038 (**40**) (EC_50_ = 0.48 μmol/L). In addition, JBJ-07-200 (**41**) (EC_50_ = 0.15 μmol/L) was obtained by replacing the hydroxyl group of JBJ-04-125-02 (**6**) with fluorine (Fig. 9), which could potentially improve membrane permeability [133]. It is highly anticipated that the further characteristic optimization and development of allosteric EGFR PROTACs will produce a valuable therapeutic strategy that will benefit more patients with EGFR-mutant disease.

**Fig. 10:**
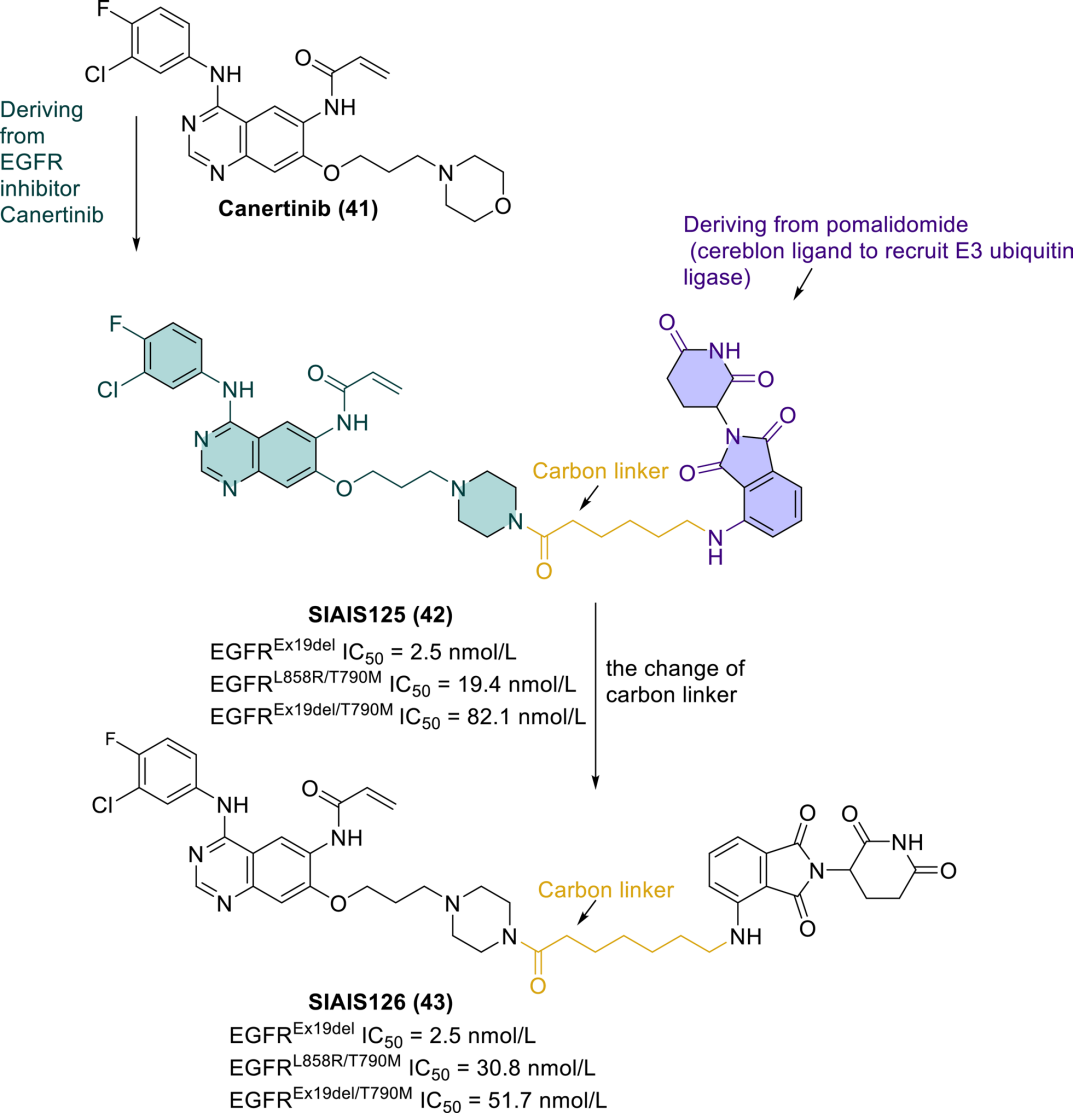
Chemical structures and EGFR inhibitory activities of novel EGFR PROTACs (through the ubiquitin/autophagy/lysosomal degradation system): the rational design of SIAIS125 (**42**) and SIAIS126 (**43**)

According to the first report by Zhao et al., EGFR degradation induced by PROTACs may be related to the autophagy pathway [134]. Qu et al. [135] demonstrated for the first time that in addition to the well-known ubiquitin/proteasome pathway, the ubiquitin/autophagy/lysosomal pathway participates in PROTAC-induced EGFR degradation. Based on the EGFR inhibitor canertinib (**41**) and the CRBN ligand pomalidomide (an E3 ubiquitin ligase ligand), researchers generated two novel EGFR PROTACs (Fig. 10), namely SIAIS125 (**42**) and SIAIS126 (**43**). These two EGFR degraders showed effective and selective antitumor activity in EGFR-TKI-resistant lung cancer cells.

#### Dual PROTACs

The basic goal of modern drug discovery is to develop efficient and selective drugs for specific targets. However, complex diseases such as cancer usually result from interactions among multiple factors, synergistic effects of multiple disease-modifying factors, the upregulation of multiple receptors, and crosstalk between signaling networks. Tumor cells readily gain drug resistance by upregulating an alternative factor or transforming the signaling pathway that promotes proliferation; therefore, treatment focused on only a single target has limitations. In addition to its issues related to drug resistance, single-target drugs also show reduced efficacy and can decrease the quality of life of patients due to side effects and tissue toxicity.

**Fig. 11:**
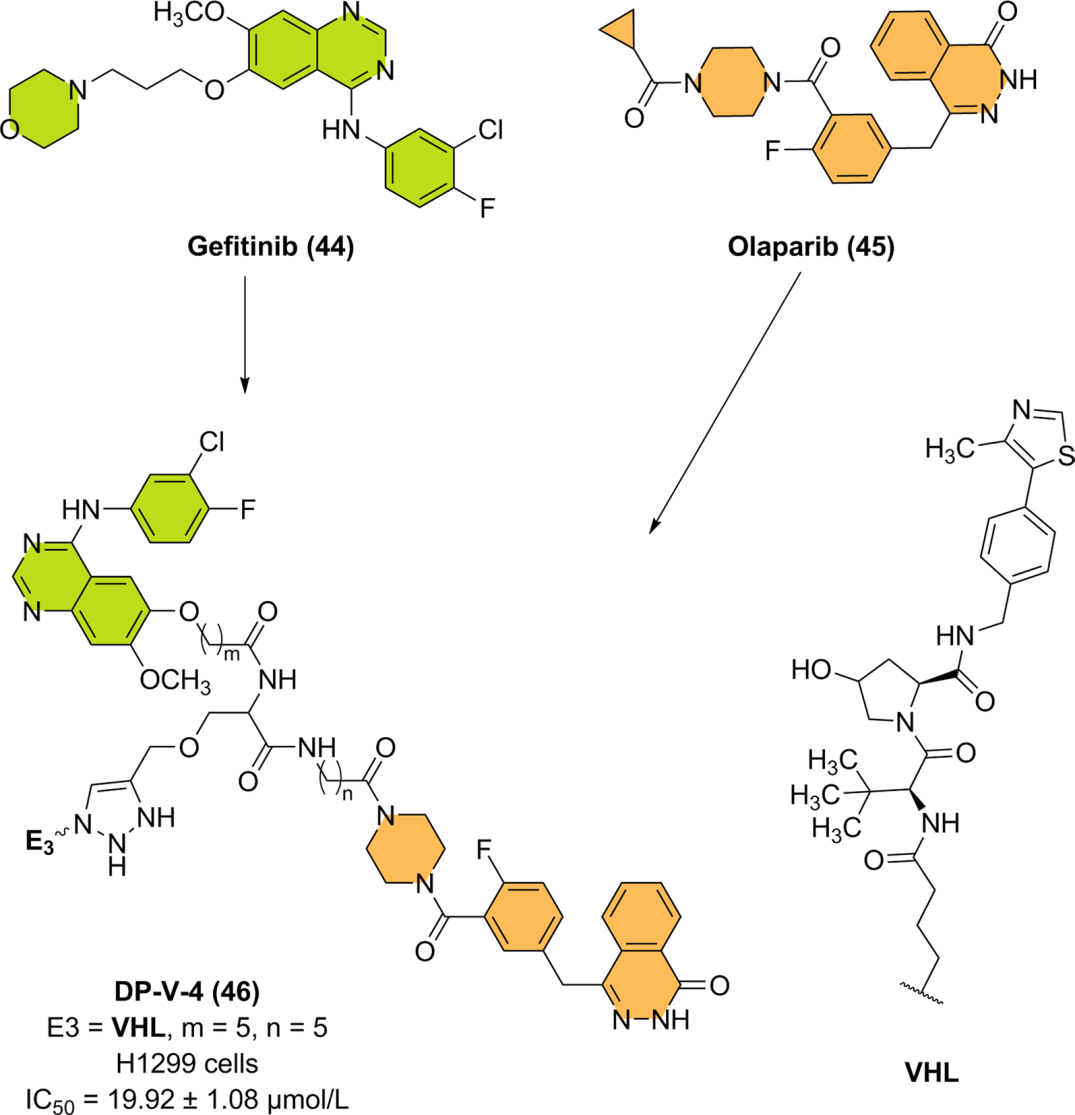
Chemical structure and antiproliferative activity of the dual PROTAC DP-V-4 (**46**) (through the ubiquitin–proteasome system)

To overcome the deficiencies of single-target drugs, single hybrid molecules fused to two or more pharmacophores have been designed to simultaneously target two or more antitumor epitopes or targets. These hybrid molecules can simultaneously modulate multiple targets or pathways and thus generally have better efficacy with fewer side effects. Based on this information and inspired by the great success of dual-targeted drugs, especially dual-specific antibodies, Professor Li et al. combined the concepts of PROTACs and dual targeting; this group used trifunctional natural amino acids as starlike core linkers to connect two independent inhibitors, gefitinib (**44**) and olaparib (**45**), that are linked to CRBN or VHL E3 ligands. The synthesized novel dual PROTACs can successfully and simultaneously degrade EGFR and poly(ADP-ribose) polymerase (PARP) in cancer cells [136]. Among the developed compounds, compound DP-V-4 (**46**) exhibited the best ability to degrade EGFR and PARP in a dose- and time-dependent manner in H1299 cells and human epidermal carcinoma A431 cells (Fig. 11). As the first successful example of a dual PROTAC, this research will inject new vitality into the field of combination therapy for cancer. Moreover, these findings will broaden the potential applications of the PROTAC method, open new fields of drug discovery, and overcome the limitations of single-target therapy against EGFR.

Another new technology is the autophagy-targeting chimera (AUTAC), a small molecule that targets protein degradation through autophagy and contains both a degradation tag (guanine derivative) and a warhead to provide target specificity; AUTACs have a wider substrate panel than the ubiquitin–proteasome system [137–139]. Therefore, there is considerable potential for the design and development of AUTAC molecules to degrade EGFR.

#### Monoclonal antibodies and ADCs

**Fig. 12:**
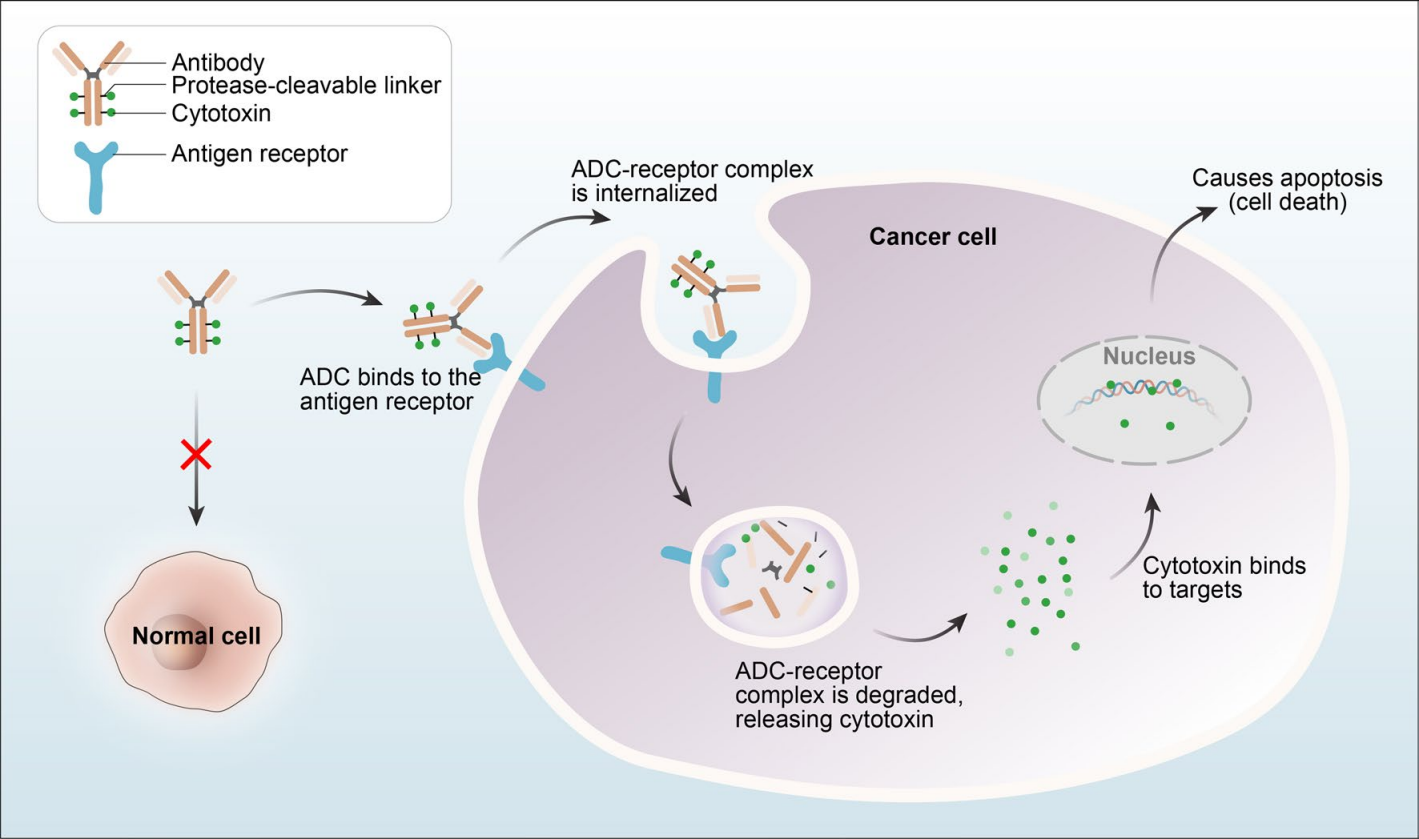
The mechanism of ADCs

For patients with EGFR-mutant disease, there are targeted therapies for tumors harboring EGFR-TKI-sensitizing mutations [140]. The EGFR monoclonal antibody can bind to the extracellular domain of EGFR to compete with EGF binding, thereby blocking downstream signaling. The variable fragment (Fv) is composed of parts of the light chain and heavy chain of the antibody and has unique antigen recognition function. The constant region (Fc) mediates innate immunity related to monoclonal antibodies, mainly by binding immune factors or cells to exert antitumor effects. These properties make antibodies a favorable approach in targeted therapy, especially in combination with other strategies. In addition, the internalization and degradation of EGFR monoclonal antibody and receptor complexes can downregulate EGFR on the surface of cancer cells. EGFR monoclonal antibodies are now standard-of-care therapies for head and neck cancer and colorectal cancer. Common EGFR monoclonal antibodies include cetuximab, necitumumab, panitumumab, matuzumab, and nimotuzumab. Antibody–drug conjugates (ADCs) are composed of three moieties: the antibody, linker, and drug (especially those with potential cytotoxicity) (Fig. 12). Antibodies are equivalent to precise arrows, and highly active cytotoxic drugs (the payload) correspond to the gunpowder on the arrows; these drugs mainly include tubulin inhibitors (monomethyl auristatin E, monomethyl auristatin F, mertansine, and ravtansine) and DNA-damaging agents (those that cause DNA double-strand breaks, DNA alkylation, DNA intercalation, and DNA cross-linking). It is difficult to effectively kill tumor cells with only cytotoxic drugs, but monoclonal antibodies alone are too inefficient. ADCs composed of both the cytotoxic drug and a monoclonal antibody represent a more powerful combination. ADCs can precisely target tumor cells by combining highly specific monoclonal antibodies with highly toxic cytotoxic drugs, thereby achieving a precise attack on EGFR-TKI-resistant cancer cells and filling the gap between antibody drugs and traditional chemotherapy drugs. The ADC approach can improve both the drug specificity and the treatment window. Being precise and efficient, ADCs have therapeutic potential across cancer types and can also induce tumor cell death via the bystander effect [141].

**Fig. 13:**
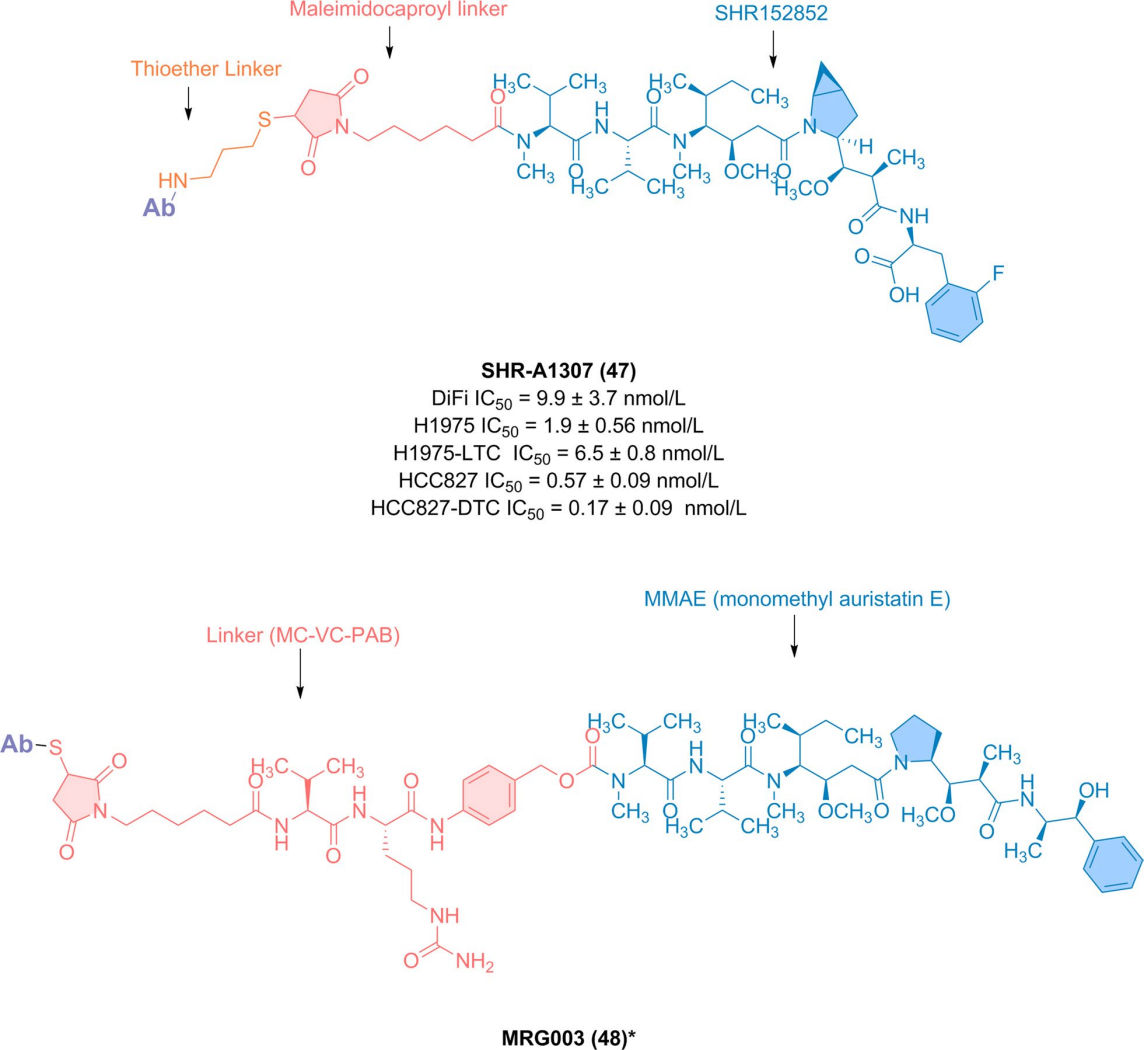
Chemical structure of ADCs targeting EGFR: SHR-A1307 (**47**) and MRG003 (**48**) (*Specific research data not disclosed)

He et al. developed a new ADC targeting EGFR, namely SHR-A1307 (**47**) (Fig. 13), for the treatment of solid tumors resistant or refractory to EGFR-targeted therapy [142]. SHR-A1307 (**47**) has intermediate ability to block EGFR affinity for hR3 and selectively binds to cancer cells expressing EGFR while avoiding inhibitory effects on normal cells. In addition to increasing stability and reducing systemic toxicity, Fc domain engineering improved the pharmacokinetics. Although less frequent drug administration may reduce toxin accumulation, effective tumor cell killing with minimal toxicity were observed. In addition, SHR-A1307 (**47**) can effectively kill cancer cells that do not respond to current EGFR inhibitors and shows low nanomolar in vitro cytotoxicity in a broad spectrum of cancer cells with different drug resistance mutations, thus providing an attractive treatment opportunity to overcome the drug resistance of patients with EGFR-overexpressing tumors.

MRG003 (**48**) [143, 144], the first EGFR ADC to enter the clinical trials in China, is composed of a humanized anti-EGFR monoclonal antibody and the tubulin inhibitor MMAE coupled through a degradable VC (Val-Cit) linker (Fig. 13). The phase I dose escalation and expansion study for patients with refractory solid tumors has been completed. Based on the results of the phase Ia and Ib clinical trials, Lepu Biosciences is currently conducting phase II clinical trials of MRG003 monotherapy in China for recurrent or metastatic advanced head and neck squamous cell carcinoma, advanced NSCLC, biliary tract cancer, and nasopharyngeal carcinoma.

#### Combination therapy strategy

Resistance to third-generation EGFR inhibitors mediated by EGFR-independent mechanisms can develop through the activation of alternative bypass pathways and abnormal downstream signal transduction closely related to tumor growth, invasion and metastasis. In the clinic, HER2 mutation, high HGF expression, and abnormal activation of MET, AXL, IGF1R and the FGFR pathway were found in patients with acquired resistance to third-generation EGFR-TKIs. Mutation or abnormal expression of EGFR signaling pathway-related genes involved in the Ras/Raf/MEK/ERK/MARK, PI3K/PDK1/Akt, PLC-γ and JAK/STAT pathways was also found. Importantly, these aberrations can coexist in the same tumor and with EGFR-TKI tertiary mutations, which are the basis for the complexity and heterogeneity of cancer evolution in response to EGFR-TKI treatment. Therefore, in combination with third-generation EGFR-TKIs, targeting important components of alternative bypass pathways (Table 3) [145–154] and downstream signal transduction pathways (Table 4) [155–164] appears to be a promising treatment strategy.

**Table 3:** Combination therapy with the bypass pathway target

Target	Representative compound*	Structure	Reference
MET	Cabozantinib		[145, 146]
MET	Crizotinib		[147]
MET	Savolitinib		[148]
FGFR	AZD4547		[149]
ALK	Lorlatinib		[150]
ALK	Brigatinib		[151]
HER2	JQ1		[152]
HER2	Trastuzumab-DM1		[153]
BRAF V600E	Encorafenib (LGX818)		[64]
AURK B	PF-03814735		[60, 154]

*Osimertinib is a representative third-generation EGFR-TKI

**Table 4:** Combination therapy with targets in downstream signaling pathways

Target	Representative compound*	Structure	Reference
MEK	Trametinib		[155, 156]
MEK	Selumetinib		[155–157]
MEK	PD0325901		[155]
AKT	Uprosertib (GSK2141795)		[158]
AKT	Capivasertib (AZD5363)		[158]
AXL	Cabozantinib		[159]
AXL	DS-1205b		[160]
AXL	Yuanhuadine (YD)		[161]
AXL	Bemcentinib (R428)		[162]
ACK1	(R)-9b		[163, 164]

*Osimertinib is a representative third-generation EGFR-TKI

#### Multitarget inhibitors

Cancer is a multifactorial disease, and single-target treatments may have poor efficacy. As clinical targeted therapy, EGFR kinase inhibitors are effective only when the cancer cells contain specific EGFR-activating mutations that alter downstream signaling [165]. Moreover, only a small proportion of patients benefit from EGFR inhibitors [2]. In addition to activating mutations at the EGFR locus that lead to drug resistance, a large number of genetic and epigenetic abnormalities may also lead to resistance to third-generation EGFR-TKIs. The emergence of intrinsic and acquired resistance requires appropriate strategies to prevent serious side effects. Combination therapy has additive or even synergistic effects, but due to various dose-limiting toxicities and drug–drug interactions caused by changes in pharmacokinetics, the simultaneous use of two or more drugs in the clinic is challenging. Therefore, as an alternative to combination therapy, drugs targeting two or more objects have a lower risk of drug–drug interactions and better pharmacokinetic and safety profiles, which helps mitigate poor patient compliance, off-target effects, and high development costs. Such treatment regimens are more flexible and can represent an effective strategy for cancer therapy [166, 167]. The effectiveness of multitarget kinase inhibitors of WT and/or mutant EGFR has been extensively studied (Table 5) [59, 168–197]. Some EGFR-mutant cell lines are sensitive to multitarget inhibition and maintain certain levels of activity, highlighting the selectivity of multitarget compounds and suggesting that multitarget inhibition can be used to circumvent acquired multidrug resistance to EGFR-targeted therapy without serious side effects.

**Table 5:** Multitarget inhibitors

Number	Target	Pharmacophores	Structure	Activity
**69** [168]	EGFR/FGFR1	Pyrimidine-2,4-diamines		EGFR^L858R/T790M^ IC_50_ = 43.1 nmol/L EGFR^WT^ IC_50_ = 1138.7 nmol/L FGFR1^WT^ IC_50_ = 17.6 nmol/L H1975 cells IC_50_ = 336.3 nmol/L
**70** [169]	EGFR/Src	Pyrimidine-4-amines		K562 cells IC_50_ = 220 nmol/L A549 cells IC_50_ = 250 nmol/L EGFR inhibition rate = 33.15% (10 µmol/L) Src inhibition rate = 72.12% (1 µmol/L)
**71** [170]	EGFR/HER4	3-Cyanoquizolines		EGFR^L858R^ IC_50_ = 419 nmol/L EGFR^WT^ IC_50_ = 2.4 nmol/L HER4 IC_50_ = 0.03 nmol/L
**72** [171]	EGFR/COX2	1,3,4-Oxadiazole scaffold		EGFR IC_50_ = 280 nmol/L COX2 IC_50_ = 170 nmol/L UO-31 cells IC_50_ = 5800 nmol/L
**73** [172]	EGFR/BRAF	Spirobenzo[h]chromene derivatives		EGFR IC_50_ = 1200 nmol/L BRAF IC_50_ = 2600 nmol/L A549 cells IC_50_ = 1780 nmol/L MCF-7 cells IC_50_ = 4090 nmol/L HT-29 cells IC_50_ = 4450 nmol/L
**74** [173]	EGFR^T790M^/ALK	Pyrimidine-2,4-diamines		ALK IC_50_ = 18 nmol/L EGFR^WT^ IC_50_ = 151 nmol/L EGFR^T790M^ IC_50_ = 2 nmol/L EGFR^L858R/T790M^ IC_50_ = 4 nmol/L DFCI032 cells IC_50_ = 170 nmol/L DFCI076 cells IC_50_ = 820 nmol/L
**75** [174]	EGFR^WT^ and mutant EGFR/ALK	Pyrimidine-2,4-diamines		EGFR^WT^ IC_50_ = 108 nmol/L EGFR^T790M^ IC_50_ = 3.9 nmol/L EGFR^L858R/T790M^ IC_50_ = 3.6 nmol/L ALK^WT^ IC_50_ = 9.8 nmol/L ALK^R1275Q^ IC_50_ = 0.82 nmol/L ALK^L1196M^ IC_50_ = 0.59 nmol/L ALK^F1174L^ IC_50_ = 0.92 nmol/L ALK^C1156Y^ IC_50_ = 1.0 nmol/L H1975 cells GI_50_ = 15 nmol/L H3112 cells GI_50_ < 0.3 nmol/L
**76** [175]	EGFR/ATX	Pyrimidine-4-amines		EGFR IC_50_ = 24.2 nmol/L ATX IC_50_ = 29.1 nmol/L A549 cells IC_50_ = 4960 nmol/L MKN-45 cells IC_50_ = 3430 nmol/L SGC cells IC_50_ = 2910 nmol/L CFs cells IC_50_ = 1490 nmol/L
**77** [176]	EGFR/AURK A	Pyrimidine-4-amines		AURK A IC_50_ = 1990 nmol/L EGFR IC_50_ = 3.76 nmol/L
**78** [177]	EGFR/IGF1R	Pyrimidine-2-amines		EGFR IC_50_ = 35.5 nmol/L EGFR^T790M^ IC_50_ = 66.0 nmol/L IGF1R IC_50_ = 52.0 nmol/L
**79** [178]	EGFR/tubulin	Pyrimidine-4-amines		EGFR IC_50_ = 30 nmol/L Tubulin assembly IC_50_ = 710 nmol/L HeLa cells IC_50_ = 1 nmol/L HT-29 cells IC_50_ = 20 nmol/L Jurkat cells IC_50_ = 1 nmol/L RS4;11 cells IC_50_ = 1 nmol/L
**80** [179]	EGFR/tubulin	Chalcones		EGFR IC_50_ = 39 nmol/L Tubulin polymerization IC_50_ = 8840 nmol/L MCF-7 cells IC_50_ = 1650 nmol/L HCT-116 cells IC_50_ = 3610 nmol/L
**81** [180]	EGFR/AKT	Chalcones		A549 cells IC_50_ = 3820 nmol/L MDA-MB-231 cells IC_50_ = 5890 nmol/L SKBR3 cells IC_50_ = 4790 nmol/L
**82** [181]	EGFR/HDACs	Pyrimidine-2-amines		EGFR^WT^ IC_50_ = 5700 nmol/L EGFR^T790M^ IC_50_ = 5000 nmol/L HDACs IC_50_ = 85 nmol/L A549 cells IC_50_ = 2190 nmol/L HeLa cells IC_50_ = 1850 nmol/L MDA-MB-231 cells IC_50_ = 600 nmol/L MDA-MB-468 cells IC_50_ = 230 nmol/L
**83** [182]	EGFR/PDGFR-β	Pyrimidine-2,4-diamines		EGFR *K*_*i*_ IC_50_ = 170 nmol/L PDGFR-β IC_50_ = 81 nmol/L
**84** [183]	EGFR/NF-κB	Quinazolines		EGFR IC_50_ = 60.1 nmol/L NF-κB IC_50_ = 300 nmol/L
**85** [184]	EGFR/c-Met	1,2,4-Oxadiazole derivate		MDA-MB-231 cells IC_50_ = 200 nmol/L A459 cells IC_50_ = 200 nmol/L PC9 cells IC_50_ = 500 nmol/L H1975 cells IC_50_ = 300 nmol/L CL68 cells IC_50_ = 400 nmol/L CL97 cells IC_50_ = 500 nmol/L
**86** [185]	EGFR^T790M^/c-Met	Pyrimidine-2-amines		EGFR^T790M^ IC_50_ = 97 nmol/L c-Met IC_50_ = 518 nmol/L
**87** [186]	EGFR/VEGFR-2	Pyrimidine-2,4-diamines		EGFR *Ki* IC_50_ = 80 nmol/L VEGFR-2 *Ki* IC_50_ = 3240 nmol/L NCI-H460 cells GI = 25% (10 µmol/L)
**88** [187]	EGFR/VEGFR-2	Quinazolines		EGFR IC_50_ = 1.0 nmol/L VEGFR-2 IC_50_ = 79.0 nmol/L HT-29 cells IC_50_ = 1760 nmol/L MCF7 cells IC_50_ = 7280 nmol/L
**89** [188]	EGFR/VEGFR-2	Quinazolines		EGFR IC_50_ = 0.69 nmol/L VEGFR-2 IC_50_ = 67.84 nmol/L
**90** [59]	EGFR/VEGFR-2	Quinazolines		EGFR IC_50_ = 2.0 nmol/L VEGFR-2 IC_50_ = 103.0 nmol/L A431 cells IC_50_ = 14.0 nmol/L H1975 cells IC_50_ = 130.0 nmol/L
**91** [189]	EGFR/VEGFR-2	Quinazolines		EGFR IC_50_ = 20 nmol/L VEGFR-2 IC_50_ = 50 nmol/L
**92** [190]	EGFR/HER2	Quinazolines		EGFR IC_50_ = 0.69 nmol/L HER2 IC_50_ = 42.1 nmol/L NCI-H1975 cells IC_50_ = 12.20 nmol/L HCC827 cells IC_50_ = 0.31 nmol/L A431 cells IC_50_ = 1.52 nmol/L MDA-MB-453 cells IC_50_ = 0.62 nmol/L
**93** [191]	EGFR/HER2	Pyrimidine-4-amines		EGFR IC_50_ = 186 nmol/L VEGFR-2 IC_50_ = 254 nmol/L
**94** [192]	EGFR/HER2	3-Cyanoquizolines		EGFR IC_50_ = 597 nmol/L IGF1R IC_50_ = 908 nmol/L A431 cells IC_50_ = 1890 nmol/L SKBR3 cells IC_50_ = 1930 nmol/L
95 [193–195]	EGFR/HER2	Pyrimidinones		EGFR IC_50_ = 60 nmol/L HER2 IC_50_ = 300 nmol/L A549 cells IC_50_ = 280 nmol/L
96 [196]	EGFR/CSK	Chalcones		EGFR IC_50_ = 11,120 nmol/L CSK IC_50_ = 5160 nmol/L
97 [197]	EGFR/CAIX	Quinazolines		EGFR^WT^ IC_50_ = 27.0 nmol/L EGFR^T790M^ IC_50_ = 9.2 nmol/L hCAII IC_50_ = 278.2 nmol/L hCAIX IC_50_ = 115.0 nmol/L A549 cells (hypoxia) IC_50_ = 2210 nmol/L A549 cells (normoxia) IC_50_ = 6450 nmol/L HeLa cells IC_50_ = 1850 nmol/L H1975 cells (hypoxia) IC_50_ = 1050 nmol/L H1975 cells (normoxia) IC_50_ = 1940 nmol/L

#### Natural products

The discovery of natural products offers new scaffolds for drug development. Natural products are an important source of compounds to overcome resistance to third-generation TKIs and provide ample possibilities for new drug discovery. Honokiol (HNK) (**98**) is a natural product purified from *Magnolia* used as a human nutritional supplement, with good tolerance and safety profiles. Many preclinical studies have shown that HNK (**98**) has potential antitumor activity against different types of cancer. Zang et al. proved that the decrease in Mcl-1 and the increase in BIM are the key mechanisms by which osimertinib induces the apoptosis of NSCLC cells with EGFR-TKI-sensitive mutations. HNK (**98**) and its derivative CAz-p (**99**) in combination with osimertinib effectively reduced the survival and induced the apoptosis of EGFR ex19del/C797S (trans) double-mutant PC-9/2 M cells and EGFR ex19del/T790M/C797S (cis) triple-mutant PC-9/3 M cells [198]. It is highly encouraging that HNK (**98**) and its derivatives may overcome clinical resistance to third-generation TKIs.

Overexpression of MCL-1 induces acquired resistance to osimertinib. Combination therapy with MCL-1 inhibitors and osimertinib is a potential strategy to overcome resistance. Bufalin (**100**) is a natural product that belongs to the class of bufadienolide analogs. A recent study found that bufalin (**100**) can reverse acquired resistance to osimertinib by inducing Ku70-mediated Mcl-1 degradation. Moreover, combined treatment with bufalin (**100**) and osimertinib triggered significant cell apoptosis and increased the levels of cleaved caspase-3 and PARP [199].

**Fig. 14:**
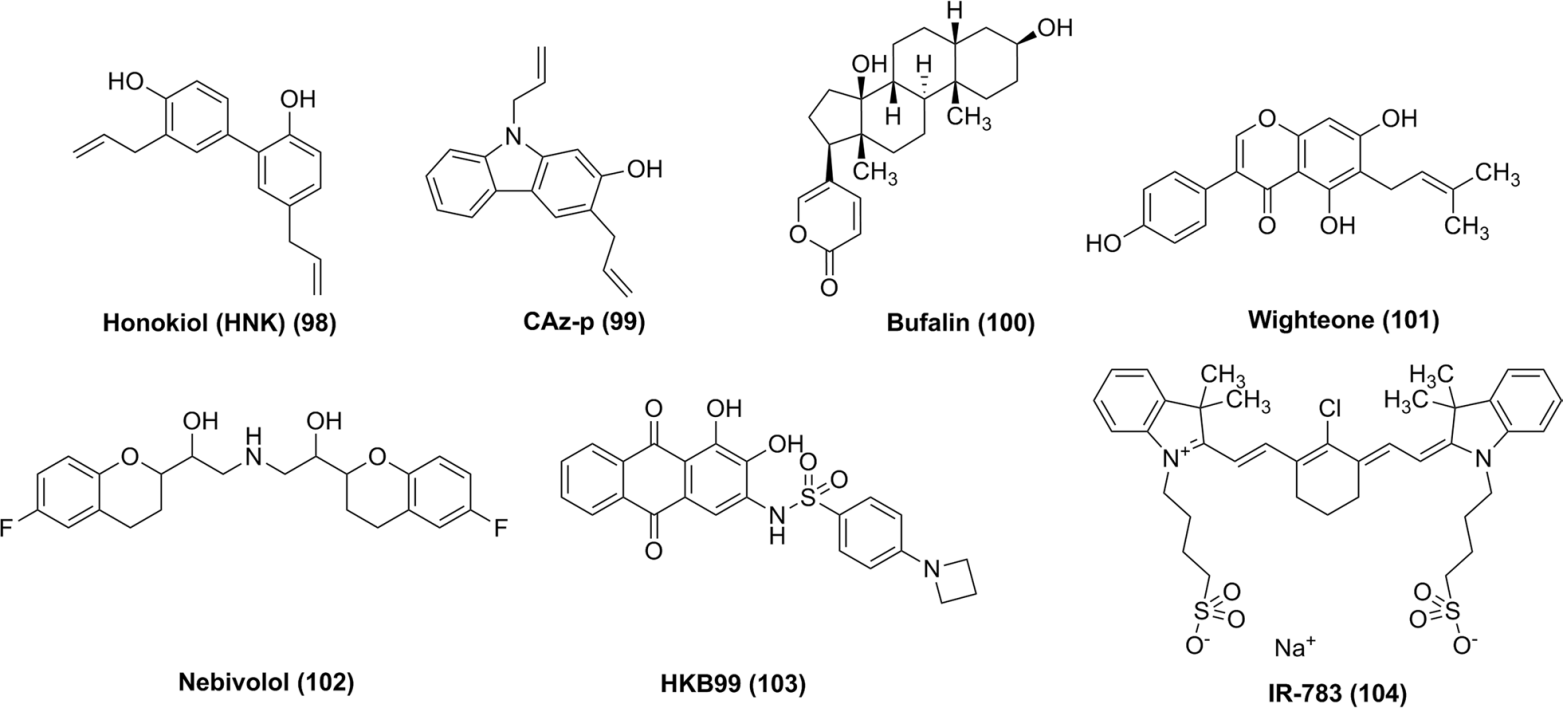
Chemical structures of natural products with their synthetic analogs and other inhibitors (no specific IC_50_ values are shown due to variations in EGFR-mutant cell line survival and apoptosis assays)

Wighteone (**101**) is a natural flavonoid compound widely found in plants. Sun et al. reported that wighteone (**101**) docks at the ATP binding site of EGFR L858R/T790M and forms two hydrogen bonds with the carbonyl group of Gln791 and the amino group of Met793, indicating that it may directly bind to EGFR L858R/T790M. Wighteone has a significant inhibitory effect on Ba/F3 and NCI-H1975 cells expressing EGFR L858R/T790M, with IC_50_ values of 1.88 μmol/L and 5.70 μmol/L, respectively [200] (Fig. 14).

## Other strategies

### EGFR degradation based on the FBXL2-Grp94-EGFR axis

Xiao’s research group found that the F-box protein Fbxl2 (an E3 ubiquitin ligase) can target EGFR and EGFR-TKI-resistant mutants for proteasome-mediated degradation independent of EGF stimulation. They also discovered that glucose regulatory protein 94 (Grp94) protects EGFR from degradation by blocking the binding of Fbxl2 to EGFR. Through virtual screening of the DrugBank database, small compounds that can bind to the Fbxo3-apag domain were scored. Nebivolol (**102**) can be placed in the dumbbell-shaped cavity of the APAG region. There are 5 amino acid residues in the center of this cavity (I331, E341, T367, T368 and F369); T367 and T368 project into the cavities of complementary shapes, forming hydrophobic interactions with the ligand. The binding affinity of the Fbxo3 protein for endogenous Fbxl2 is greatly reduced when these five amino acids are mutated individually or in combination. Data suggest the potential of nebivolol (**102**) as a small molecule that can disrupt the Fbxo3–Fbxl2 interaction. Increasing Fbxl2 levels with nebivolol (**102**) (Fig. 14) in combination with osimertinib or a Grp94 inhibitor (ganetespib) to target the FBXL2-Grp94-EGFR axis and thus destabilize EGFR is a possible therapeutic strategy to overcome resistance to third-generation EGFR-TKIs [201].

#### AKR1B1 inhibitors

Zhang et al. discovered that aldehyde ketone reductase family 1 member B1 (AKR1B1) interacts with STAT3 and activates the cystine transporter solute carrier family 7 member 11 (SLC7A11), which in turn leads to enhanced cystine uptake, glutathione synthesis flux, clearance of reactive oxygen species (ROS), protection against cell death, and EGFR-TKI resistance. The use of selective inhibitors (including the clinically approved anti-diabetic drug epalrestat) to inhibit AKR1B1 can restore the sensitivity of drug-resistant cell lines to EGFR-TKIs and delay drug resistance in mice harboring xenografted tumors derived from lung cancer patients [202].

#### PGAM1 inhibitors

Phosphoglycerate mutase 1 (PGAM1) is an important enzyme in the glycolysis pathway and is related to tumor cell metastasis [203]. HKB99 (**103**) (Fig. 14) is an allosteric inhibitor of PGAM1 that significantly inhibits the growth and metastasis of NSCLC by affecting the metabolic activity and nonmetabolic functions of PGAM1 [204]. The docking model of the PGAM1-HKB99 complex shows that HKB99 (**103**) binds to the allosteric site of the adjacent substrate-binding pocket of PGAM1, thereby inhibiting the conversion of 3-PG to 2-PG and significantly reducing the metabolic activity of PGAM1. In addition, HKB99 (**103**) can allosterically bind to PGAM1, weaken the interaction between PGAM1 and ACTA2, and inhibit the growth and metastasis of erlotinib-resistant lung cancer cells [205, 206]. Therefore, PGAM1 is a metabolic enzyme that may overcome EGFR-TKI resistance.

#### Nonoverlapping allosteric pockets—the X-Pocket

Qiu et al. revealed the underlying mechanism of reverse allosteric communication in dual-targeted therapy. Allosteric sites can be affected by orthomorphic drugs. The nonoverlapping allosteric pocket X-Pocket was discovered in EGFR mutants; this pocket is mainly composed of nonconserved residues, including the hot spots K867, S895, and K960, that can cooperate with traditional TKIs [207]. It is a promising target for the design of selective conformationally restricted drugs, with great potential in terms of affinity, efficacy, and selectivity.

#### DZ-SIM inhibitors

In addition, researchers found that a group of near-infrared heptamethine carbocyanine (DZ) fluorescent dyes, the prototype of which is heptamethylamine carbocyanine dye (IR-783) (**104**) (Fig. 14), have tumor-targeting activity through differentially expressed organic anion transport peptides on cancer cells [208]. This group of organic dyes can specifically deliver therapeutic payloads to tumor cells in the form of chemical conjugates. DZ-SIM was preliminarily synthesized; SIM specifically targets 3-hydroxy-3-methylglutaryl-CoA reductase (HMGCR) in the endoplasmic reticulum. After specific uptake by tumor cells, DZ-SIM was enriched in subcellular organelles (including mitochondria and lysosomes). NSCLC cells were killed by mitochondrial damage, which mainly led to cytochrome C release into the cytoplasm, thereby activating the caspase-3-dependent apoptosis cascade. DZ-SIM inhibited the formation of cancer cell colonies resistant to first-generation (H1650 and H1975) and third-generation EGFR-TKIs (PC9AR), and most IC_50_ values were lower than 10 μmol/L. DZ-SIM represents a promising new therapy to overcome drug resistance in patients with EGFR-mutant disease.

#### Selection of individualized combination therapy

For patients who experience SCLC transformation, chemotherapy after the development of osimertinib resistance is an option. Research has shown that patients with transformation to SCLC have higher response rates to etoposide, cisplatin, and paclitaxel. For patients with unclear resistance mechanisms, chemotherapy is still a treatment option. If the patient is asymptomatic or has symptomatic local progression, osimertinib can be combined with local treatment according to National Comprehensive Cancer Network (NCCN) guidelines. Carboplatin, paclitaxel, bevacizumab, and atezolizumab (anti-PD-L1 antibody) are also options for patients who experience systemic progression after osimertinib treatment [209]. Whether chemotherapy can delay the development of resistance to third-generation EGFR-TKIs remains unknown. A study on osimertinib with or without chemotherapy as first-line therapy for patients with EGFR-mutant NSCLC is currently recruiting (NCT04035486) [210].

For most patients, the PD-1/PD-L1 pathway is not the sole rate-limiting factor for antitumor immunity, and blocking the PD-1/PD-L1 axis is insufficient to activate an effective antitumor immune response [211]. Strategies that lead to acquired EGFR-TKI resistance, such as HGF, MET amplification, and EGFR T790M, also promote immune escape in lung cancer by upregulating the expression of PD-L1. Many combination strategies, including α-PD-1/PD-L1 plus chemotherapy, radiotherapy, angiogenesis inhibitors, targeted therapy, other ICIs, agonists of the costimulatory molecule, stimulator of interferon gene agonists, epigenetic modulators, or metabolic modulators, have been confirmed to have superior antitumor efficacy and a higher response rate. The immunomodulatory effect of chemotherapy suggests that it might be a suitable partner for combination with α-PD-1/PD-L1 to achieve rapid and long-term cancer control. During the KEYNOTE series of clinical trials (such as KEYNOTE-021, KEYNOTE-189, and KEYNOTE-407), pembrolizumab combined with standard chemotherapy led to better overall survival (OS) and progression-free survival (PFS) in NSCLC patients and has been approved by the FDA as first-line treatment for advanced nonsquamous NSCLC [212, 213]. In addition, the National Medical Products Administration (NMPA) approved sintilimab plus gemcitabine and platinum as first-line treatment for advanced squamous NSCLC based on the results of ORIENT-12 [214]. In addition to α-PD-1-based approaches, α-PD-L1-based chemoimmunotherapy has also attracted intense attention. The IMpower150 trial was the pioneer of this series of studies, and the FDA-approved atezolizumab plus bevacizumab, paclitaxel, and carboplatin as first-line treatment for advanced nonsquamous NSCLC [215]. Subsequently, the FDA-approved atezolizumab plus nab-paclitaxel and carboplatin for nonsquamous NSCLC (based on the results of IMpower130). Radiotherapy can also induce immunogenic cell death and enhance the antitumor immune response. The results of a phase 1 study showed that α-PD-1/PD-L1 plus chemoradiotherapy was tolerable in advanced NSCLC (NCT02621398), with promising clinical outcomes. In multiple clinical studies, such as IMpower150, angiogenesis inhibitors enhanced the efficacy of α-PD-1/PD-L1 [216]. Moreover, dual immune checkpoint blockade or costimulatory molecule agonists plus α-PD-1/PD-L1 are also promising strategies. To date, the FDA has approved ipilimumab plus nivolumab for NSCLC and melanoma, among others. Agonists targeting costimulatory pathways such as CD27/CD70, CD40/CD40L, and 4-1BB/4-1BBL could also enhance T-cell activity and restore the antitumor immune response. However, bispecific/bifunctional antibodies simultaneously block two molecules and thus have a strategic advantage over combination therapy. For example, in the phase 1 NCT03710265 trial, SHR-1701 (TGF-β × PD-L1 bifunctional antibody) showed encouraging antitumor activity [217].

Given the heterogeneity of mutations across patients, the selection of individualized combination treatment strategies could improve outcomes and mitigate treatment resistance.

## Discussion and future perspectives

EGFR is an important target on tumor cells that promotes mitosis and transformation. It is overexpressed in many diseases and is particularly related to the occurrence and development of cancer [3, 7, 8]. Tumors often have prominent genomic and transcriptional heterogeneity that is closely related to EGFR-TKI resistance [40, 218]. Studies have shown that drug resistance can develop through EGFR-dependent and EGFR-independent mechanisms [24, 219]. The emergence of resistance to third-generation EGFR-TKIs limits the clinical benefits for patients, thus necessitating the further development of more effective strategies.

To date, fourth-generation EGFR-TKIs show prominent antitumor activity. Recent research has shown that fourth-generation inhibitors targeting allosteric sites and ATP-competitive sites of EGFR can achieve remarkable results against EGFR L858R/T790M and C797S. In addition to fourth-generation EGFR-TKIs, combination treatments, monoclonal antibodies, and bispecific antibodies are significantly contributing to the treatment of tumors harboring the C797S mutation. While the C797S mutation is only one of the numerous drug resistance mechanisms, it is necessary to overcome other mutations by designing and developing new noncovalent ATP-competitive inhibitors that form hydrogen bonds with mutated residues in the EGFR ATP pocket (such as Lys745 and Asn842). The rational design of selective EGFR inhibitors that bind to both the ATP and allosteric sites of the EGFR kinase domain, that is, adding allosteric inhibitor elements to the compound skeleton at the ATP binding site, will help optimize and improve the mutation selectivity of compounds and lead to the identification of small molecules with good kinase inhibitory activity. However, the cellular activity of such compounds needs to be further improved, and future research directions should focus on the structural optimization of current lead compounds to obtain EGFR inhibitors with better mutation selectivity. Targeted protein degradation technology provides a new research direction for overcoming resistance to third-generation EGFR inhibitors. Considering the significance of overcoming allosteric hindrance by triple-mutant EGFR, allosteric EGFR degraders were developed. In addition, dual PROTACs have emerged in the field of cancer combination therapy; dual PROTACs can be designed with two targets, such as tumor immune targets plus adjuvant immune targets or energy metabolism targets and epigenetic targets plus antiapoptotic targets, to further overcome resistance of third-generation EGFR inhibitors and provide a better curative effect. Of course, the larger molecular weight of dual PROTACs will affect their druggability and pharmacokinetics, but perhaps nanodrug delivery systems can be utilized to improve drug absorption or optimized by simplifying the inhibitor moiety and maintaining the minimum pharmacophore. In addition, ADCs containing a small-molecule cytotoxic compound and a monoclonal antibody targeting a cancer target have attracted attention. The ADC MRG003 has entered clinical trials with great development and application prospects. The activation of alternative pathways and histological transformation are important mechanisms of resistance to third-generation EGFR inhibitors. The combined use of third-generation inhibitors and related pathway blockers is another important approach. To prevent the toxicity and side effects of multidrug combinations, drugs with multiple pharmacological activities were developed and proven to have more advantages than combination therapy. Multitarget kinase drugs have become a favorable choice due to their attractive pharmacokinetic characteristics and safety profiles. Natural compounds have received much research attention due to their potential antitumor effects. Based on the molecular mechanism of inhibition, natural compounds can be modified to provide new insights for effectively overcoming resistance to third-generation EGFR-TKIs. Last but not least, the discovery of EGFR degraders based on the FBXL2-Grp94-EGFR axis, AKR1B1 and PGAM1 inhibitors, DZ-SIM, and the nonoverlapping allosteric pocket X-Pocket provides promising support for the further development of strategies to overcome resistance to third-generation EGFR inhibitors. The mechanism of resistance to third-generation EGFR-TKIs is very complex, is impacted by EGFR mutations, and differs among patients and tumor sites. Thus, next-generation sequencing (NGS) of blood-based circulating tumor DNA (ctDNA) or tissue samples to elucidate the resistance mechanism will be valuable for guiding future therapeutic approaches and for clinical research on novel combination therapies to overcome drug resistance. Moreover, individualized combination treatment strategies could also improve treatment efficacy and mitigate treatment resistance.

EGFR is a verified target for antitumor therapy in a broad spectrum of cancers. Facilitated by versatile strategies in the field of medicinal chemistry, better approaches are anticipated for overcoming the hurdle of drug resistance to provide new hope for patients.

## Conclusion

As a crucial “controller” that is related to the inhibition of tumor cell proliferation, angiogenesis, invasion, metastasis, and apoptosis, EGFR actively participates in malignant disease progression. However, the intrinsic and acquired resistance in primary and recurrent cancer which is mediated by EGFR mutations after target treatment leads to difficult therapeutic. Understanding the complex resistance mechanisms of EGFR-TKIs and developing potential strategies to combat it could be of potential interest for improving the individual therapeutic strategies for cancer.

## Data Availability

The material supporting the conclusion of this review has been included within the article.

## Abbreviations

ACK1: Activated Cdc42-associated kinase 1
ADC: Antibody drug conjugates
AKR1B1: Aldehyde–ketone reductase family 1 member B1
A-loop: Activation loop
ATP: Adenosine triphosphate
AURK: Aurora kinases
AUTAC: Autophagy-targeting chimera
Bcl-2: B cell lymphoma-2
BRAF: V-RAF murine sarcoma viral oncogene homolog B1
CCND: Cyclin D
CCNE1: Cyclin E1
CDK4/6: Cyclin-dependent kinase 4/6
cIAP1: Cellular inhibitor of apoptosis protein 1
CRBN: Cereblon
DFG: Asp-Phe-Gly
DZ-SIM: DZ-SIMvastatin
EGF: Epidermal growth factor
EGFR: Epidermal growth factor receptor
EGFR-TKIs: Epidermal growth factor receptor tyrosine kinase inhibitors
EMT: Epithelial–mesenchymal transformation
EMT-TFs: EMT-induced transcription factors
ERC1: Excision repair cross-complementation 1
FGF: Fibroblast growth factor
FGFR: Fibroblast growth factor receptor
Fv: Variable region fragment
Grp94: Glucose regulatory protein 94
HB-EGF: EGF-like growth factor
HMGCR: 3-Hydroxy-3-methylglutaryl-CoA reductase
HNK: Honokiol
ICIs: Immune checkpoint inhibitors
IGF1R: Insulin-like growth factor receptor 1
LUAD: Lung adenocarcinoma
MDM2: Mouse double minute 2
NCCN: National Comprehensive Cancer Network
NSCLC: Non-small cell lung cancer
NTRK1: Neurotrophic tyrosine receptor kinase 1
PARP: Poly(ADP-ribose) polymerase
PGAM1: Phosphoglycerate mutase 1
PIK3CA: Phosphatidylinositol 4,5-bisphosphate 3-kinase catalytic subunit
PROTAC: Proteolysis-targeting chimera
PTEN: Phosphatase and tensin homolog
ROS: Reactive oxygen species
RTK: Receptor tyrosine kinase
SCLC: Small cell lung cancer
SLC7A11: Solute carrier family 7 member 11
SPP1: Secreted phosphoprotein 1
STAT: Signal sensor and transcription activator
TACC3: Transforming acid helix protein 3
TGF: Transforming growth factor
TMP3: Thrombopoietin mimetic peptide 3
VHL: Von Hippel–Lindau
WT: Wild-type
WT-EGFR: Wild-type EGFR
